# SARS-CoV-2 variants divergently infect and damage cardiomyocytes in vitro and in vivo

**DOI:** 10.1186/s13578-024-01280-y

**Published:** 2024-08-02

**Authors:** Bobo Wing-Yee Mok, Maxwell Kwok, Hung Sing Li, Lowell Ling, Angel Lai, Bin Yan, Cherie Tsz-Yiu Law, Chui Him Yeung, Anna Jinxia Zhang, Rachel Chun-Yee Tam, Anja Kukic, Conor J. Cremin, Yajie Zhang, Teng Long, Zhisen Kang, Ruibang Luo, Kam Tong Leung, Albert M. Li, Grace Lui, Stephen Kwok-Wing Tsui, Jasper Fuk-Woo Chan, Kelvin Kai-Wang To, Paul K. S. Chan, Bryan P. Yan, Honglin Chen, Ellen Ngar-Yun Poon

**Affiliations:** 1 State Key Laboratory for Emerging Infectious Diseases, Department of Microbiology, School of Clinical Medicine, Li Ka Shing Faculty of Medicine, The University of Hong Kong, Hong Kong, SAR China; 2 Centre for Virology, Vaccinology and Therapeutics, Hong Kong Science and Technology Park, Hong Kong, SAR China; 3 Department of Medicine and Therapeutics, The Chinese University of Hong Kong, Hong Kong, SAR China; 4 Hong Kong Hub of Paediatric Excellence (HK HOPE), The Chinese University of Hong Kong, Hong Kong, SAR China; 5 Department of Paediatrics, The Chinese University of Hong Kong, Hong Kong, SAR China; 6 Department of Anaesthesia and Intensive Care, The Chinese University of Hong Kong, Hong Kong, SAR China; 7 Heart and Vascular Institute, The Chinese University of Hong Kong, Hong Kong, SAR China; 8 Department of Computer Science, The University of Hong Kong, Hong Kong, SAR China; 9 The School of Biomedical Sciences, The Chinese University of Hong Kong, Hong Kong, SAR China; 10 Faculty of Medicine, The Chinese University of Hong Kong, Hong Kong, SAR China; 11 Department of Microbiology, The Chinese University of Hong Kong, Hong Kong, SAR China; 12 Centre for Cardiovascular Genomics and Medicine, Lui Che Woo Institute of Innovative Medicine, The Chinese University of Hong Kong, Hong Kong, SAR China; 13 Ministry of Education Key Laboratory for Regenerative Medicine, The Chinese University of Hong Kong, Hong Kong, SAR, China

**Keywords:** SARS-CoV-2, COVID-19, Cardiac infection, Omicron, Cardiomyocytes, Heart

## Abstract

**Background:**

COVID-19 can cause cardiac complications and the latter are associated with poor prognosis and increased mortality. SARS-CoV-2 variants differ in their infectivity and pathogenicity, but how they affect cardiomyocytes (CMs) is unclear.

**Methods:**

The effects of SARS-CoV-2 variants were investigated using human induced pluripotent stem cell-derived (hiPSC-) CMs in vitro and Golden Syrian hamsters in vivo.

**Results:**

Different variants exhibited distinct tropism, mechanism of viral entry and pathology in the heart. Omicron BA.2 most efficiently infected and injured CMs in vitro and in vivo*,* and induced expression changes consistent with increased cardiac dysfunction, compared to other variants tested. Bioinformatics and upstream regulator analyses identified transcription factors and network predicted to control the unique transcriptome of Omicron BA.2 infected CMs. Increased infectivity of Omicron BA.2 is attributed to its ability to infect via endocytosis, independently of TMPRSS2, which is absent in CMs.

**Conclusions:**

In this study, we reveal previously unknown differences in how different SARS-CoV-2 variants affect CMs. Omicron BA.2, which is generally thought to cause mild disease, can damage CMs in vitro and in vivo. Our study highlights the need for further investigations to define the pathogenesis of cardiac complications arising from different SARS-CoV-2 variants.

**Supplementary Information:**

The online version contains supplementary material available at 10.1186/s13578-024-01280-y.

## Background

Since severe acute respiratory syndrome coronavirus 2 (SARS-CoV-2) was first detected in Wuhan in 2019, multiple variants of this virus have emerged, some of which have been declared as variants of concern by the World Health Organisation due to their distinct infectivity, pathogenicity and immune evasion [1]. The Delta and Omicron variants have caused significant waves of transmission in humans across the world. The Omicron variant was first reported in Botswana and South Africa. The original Omicron BA.1 and BA.2 variants, and subsequent more transmissible Omicron sub-lineages, have since become the predominant strains in many countries [2, 3]. The remarkable transmissibility of the Omicron variants is commonly attributed to extensive amino acid substitutions in the spike protein responsible for viral entry, leading to altered tropism for infection and immune evasion [4–7]. While earlier variants such as Delta require the transmembrane serine protease 2 (TMPRSS2) for activation, Omicron infection can occur independently of this protein, allowing Omicron to infect cells that lack TMPRSS2 [4–6, 8, 9]. Thus it is speculated that Omicron has acquired different tissue tropism and altered pathogenesis compared to other variants. Much of the research has focused on cells in the respiratory system [4–6]. The infectivity and damage of different variants in non-respiratory cells such as cardiomyocytes (CMs) are not clearly understood.

Although Coronavirus disease 2019 (COVID-19) is primarily considered a respiratory disease, cardiovascular complications are frequently detected in patients with COVID-19 and are associated with poor prognosis [10, 11]. Survivors of COVID-19 are associated with a substantial risk of adverse cardiovascular disorders such as dysrhythmias, inflammatory heart disease, ischemic heart disease, and heart failure long after acute infection [12]. While risks of cardiovascular outcomes increase according to the severity of acute infection, elevated risks were evident even among non-hospitalised patients with mild disease [12]. How COVID-19 damages the heart is not clear [13], but multiple mechanisms have been proposed including hypoxia-induced cardiac damage [14], acute inflammation and myocarditis [15] and direct viral infection of CMs [16–20]. Various groups have demonstrated that SARS-CoV-2 can infect CMs and induce cytopathic effects [16–23], but most utilised earlier strains of SARS-CoV-2 [16, 20, 21]. The effect of different variants on CMs remains elusive.

We hypothesise that CMs, which do not express TMPRSS2, are more susceptible to Omicron variants, which do not require this protein for infection, than the earlier Delta variant. Using human induced pluripotent stem cells-derived cardiomyocytes (hiPSC-CMs) and the Golden Syrian Hamster as models, we examined the effect of SARS-CoV-2 variants in CMs. Our results showed that SARS-CoV-2 variants divergently infect and damage CMs in vitro and in vivo. Omicron BA.1 and BA.2 could efficiently infect hiPSC-CMs via endocytosis in a TMPRSS2-independent manner, while Delta minimally infected these cells. CM infection was associated with significant cytopathic damage and transcriptomic alterations, and these were most severe in hiPSC-CMs infected by Omicron BA.2. Our results show that although Omicron BA.2 induces a mild phenotype in the respiratory system, it can directly injure CMs in vitro and in vivo.

## Materials and methods

### Human iPSC culture and cardiac differentiation

Human iPSC line AICS-0060-027 (Allen Cell Collection) was used in our experiments. The maintenance of hiPSC culture and cardiac differentiation were performed as previously described [24]. Human iPSC-CMs were maintained for at least 40 days before use in experiments, > 90% cells were positive for cardiac troponin T as confirmed by flow cytometry [25, 26].

### Viral culture

The SARS-CoV-2 variants, Delta (GISAID: EPI_ISL_3221329), Omicron BA.1 (GenBank: OM212472) and Omicron BA.2 (GISAID: *EPI_ISL_9845731*) were isolated from specimens obtained from three laboratory-confirmed COVID-19 patients [8]. All experiments involving SARS-CoV-2 viruses were conducted in a Biosafety Level-3 laboratory.

### Infection of hiPSC-CMs

Human iPSC-CMs were infected at a multiplicity of infection (MOI) of 1 unless otherwise stated. Human iPSC-CMs were fixed with 4% paraformaldehyde or lysed with Trizol reagent (ThermoFisher Scientific, Waltham, MA) at 24and 48 h post-infection (hpi). Infection of human iPSC-CMs at an MOI of 0.1 was performed for determination of viral replication kinetics, and an MOI of 1 for drug treatment assays with camostat mesylate (Abcam), bafilomycin A1 (MedChemExpress) and E64d (MedChemExpress). The culture supernatants were collected at 24, 48, and 72 hpi for viral replication kinetics, and 48 hpi for drug treatment assays.

### Hamster study

Golden Syrian hamsters (8–10 weeks, male) were anaesthetised with ketamine (150 mg/kg) and xylazine (10 mg/mg) via intraperitoneal injection and then intranasally challenged with 10^4^ PFU (50 µl) of viruses or with PBS mock control, and were sacrificed by intraperitoneal injection of pentobarbital at 200 mg/kg 2 or 7 days post-infection (dpi) for expression and histopathological analyses as previously described [27].

### Ethics

The animal study was approved by the Committee on the Use of Live Animals in Teaching and Research of the University of Hong Kong (CULATR 5512-20).

### Statistics

GraphPad PRISM 8 (GraphPad Software, San Diego CA, USA) was used for statistical analysis. Data sets were analysed with Student’s T-test, one-way ANOVA or two-way ANOVA followed by Tukey’s multiple comparison test, presented as mean ± SEM (standard error of the mean). Differences with p-value less than 0.05 were considered statistically significant.

Please see Additional file 1, supplementary methods for detailed methodology. List of primers and antibodies used are shown in Additional file 2, Table S1 and Additional file 3, Table S2 respectively.

## Results

### Omicron BA.1 and BA.2 more readily infect hiPSC-CMs than Delta

**Fig. 1:**
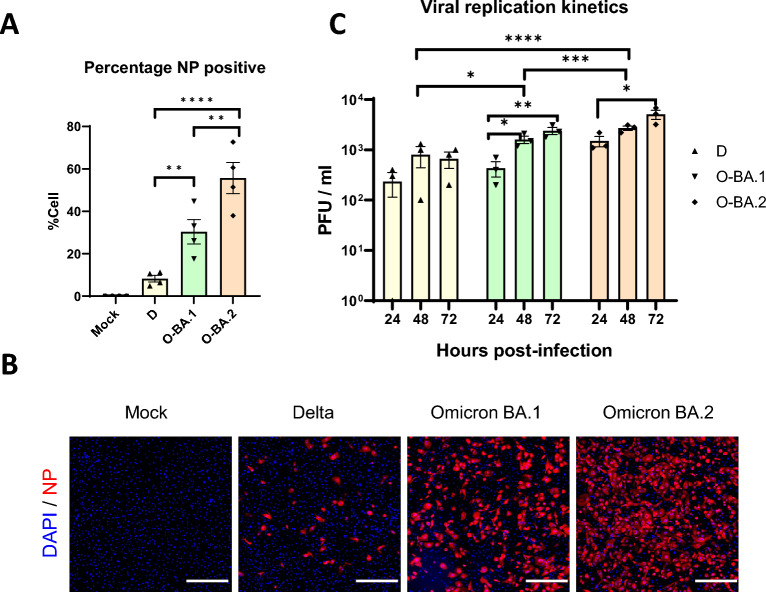
Delta, Omicron BA.1, and BA.2 differentially infect hiPSC-CMs. Human iPSC-CMs were infected with Delta (D), Omicron BA.1 (O-BA.1) or BA.2 (O-BA.2). **A** The cells were infected at a multiplicity of infection (MOI) of 1 and were immunostained for virally-encoded nucleocapsid protein (NP) at 24 h post infection (hpi). Graph shows the percentage of NP^+^ hiPSC-CM, n = 4. **B** Fluorescence images of infected hiPSC-CMs with MOI of 1 at 24 hpi, NP in Red, DAPI in blue. **C** Replication of SARS-CoV-2 was determined by infecting iPSC-CMs at an MOI of 0.1. The viral titres of supernatant collected at 24, 48, and 72 hpi were determined by plaque assay, n = 3. Data are presented as mean ± SEM, and n refer to biological replicates. Statistical significance was calculated using **A** one-way ANOVA or **C** two-way ANOVA with Tukey’s multiple comparisons test. *p < 0.05, **p < 0.01, ***p < 0.001, ****p < 0.0001. Scale bar = 500 μm

We investigated the infection and pathogenesis of SARS-CoV-2 using our in vitro hiPSC-CM model [24]. We focused on 3 (sub)-variants, Delta, Omicron BA.1 and BA.2, which have caused major global epidemics. To determine if different variants differentially infect hiPSC-CMs, these cells were infected with Delta, Omicron BA.1 or BA.2 at an MOI of 1, and assayed for the presence of virally-encoded nucleocapsid protein (NP).  Immunofluorescence staining revealed intense NP staining in the cytoplasm in hiPSC-CMs at 24 h post infection (hpi) (Fig. 1A and B). At 24 hpi, Omicron BA.2 infected cultures contained the highest proportion of NP^+^ cells (55.7 ± 7.3%), and this is significantly higher than those of Omicron BA.1 (30.4 ± 5.7%) and Delta (8.3 ± 1.6%) cultures (Fig. 1A and B). Similar trends were seen at 48 hpi and at a different MOI of 0.1 (Additional file 4, Fig. S1).

We next evaluated viral replication by measuring infectious particles in the supernatant of infected cultures. Omicron BA.1 and BA.2, but not Delta showed a significant increase in viral titre over time, indicating viral replication (Fig. 1B). Highest viral titre was detected in hiPSC-CMs infected with Omicron BA.2, followed by BA.1 and Delta (Fig. 1B). Of note, signs of degeneration were evident starting from 24 to 48 hpi and were particularly severe in Omicron BA.2 cultures, which may negatively impact upon viral replication.

### Delta and Omicron infection produce mitochondrial and structural alterations but Omicron BA.2 induce the most severe phenotype

**Fig. 2:**
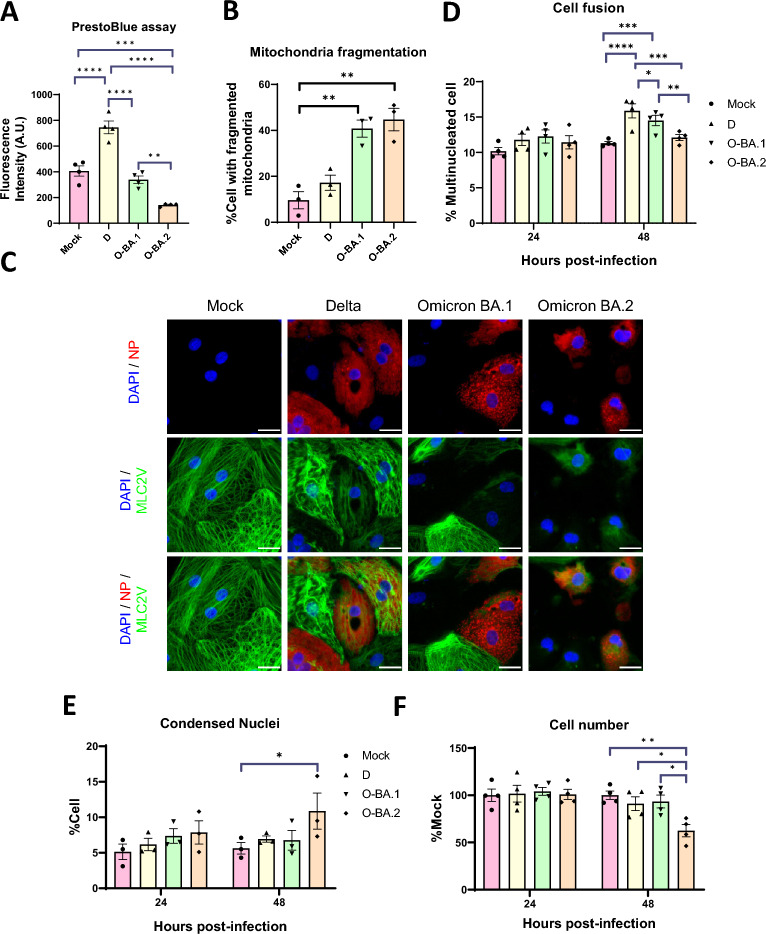
Omicron BA2 induces more severe damage in hiPSC-CMs. Human iPSC-CMs were infected with Delta (D), Omicron BA.1 (O-BA.1) or BA.2 (O-BA.2) for 48 h at MOI of 1. **A** Mitochondrial redox activity was measured using the PrestoBlue assay, n = 4. **B** The percentage of hiPSC-CMs with mitochondrial fragmentation was measured, n = 3. **C** Fluorescence images of hiPSC-CMs showing MLC2V-eGFP in green, NP in red, DAPI nuclear staining in blue. Representative images of 3 batches of cells are shown. **D** The percentage of hiPSC-CMs with more than one nuclei, n = 4. **E** The percentage of hiPSC-CMs with condensed nuclei, n = 3. **F** Cell number was normalised to that of mock infection control, n = 4. Data are presented as mean ± SEM, and n refer to biological replicates. Statistical significance was calculated using the one-way ANOVA with Tukey’s multiple comparisons test. *p < 0.05, **p < 0.01, ***p < 0.001, ****p < 0.0001. Scale bar = 25 μm

We next explored the consequences of infection using our hiPSC-CM model. Previous work has shown that SARS-CoV-2 can damage the mitochondria [28]. To test if the variants differentially affect mitochondrial function, we measured the redox potential of infected hiPSC-CM cultures at 48 hpi. Redox potential is primarily driven by the proton gradient in the mitochondria and maybe used as a surrogate measure of mitochondrial metabolism (Fig. 2A). Omicron BA.2 significantly suppressed redox potential by 63.5 ± 5.2%, while Omicron BA.1 only induced modest and non-significant reduction. Delta unexpectedly increased redox potential.

SARS-CoV-2 has been shown to perturb mitochondrial quality control [29], thus we evaluated the mitochondrial integrity of hiPSC-CMs infected with the variants. The mitochondria were visualised by immunofluorescence staining using anti-TOM20 antibody, a commonly used marker of this organelle (Fig. 2B and Additional file 5, Fig. S2A and B). Control, mock-infected hiPSC-CMs had predominantly healthy, elongated mitochondria. Conversely, a significantly increased proportion of Omicron BA.1 and 2 infected cultures exhibited unhealthy, fragmented mitochondria (43.1 ± 3.5, 46.5 ± 3.9%) vs control (8.8 ± 2.8%) at 48 hpi, while Delta did not induce significant changes.

Another prominent feature of SARS-CoV-2 infection is sarcomere breakdown, which can potentially disrupt cardiac contraction [21]. Our hiPSC line express the sarcomeric protein MLC2V endogenously tagged to the enhanced green fluorescent protein (eGFP) reporter [24], which enables us to examine sarcomere integrity by monitoring MLC2V-eGFP signal (Fig. 2C). Control, mock infected cells had well organised sarcomeres, with striated MLC2V-eGFP signal localised to the A-band of CMs. Conversely, MLC2V-eGFP signal was disorganised and weaker among infected cultures irrespective of the variants.

SARS-CoV-2 has been shown to damage respiratory cells by inducing syncytia formation [5, 30]. The higher pathogenicity of Delta relative to Omicron is partly attributed to increased fusogenicity of the former [5]. To test if the variants can similarly induce syncytia formation in CMs, we quantified the number of multi-nucleated cells after infection (Fig. 2D). Control hiPSC-CMs contained a small proportion of multi-nucleated cells, which is a normal feature of human CMs. Infection by Delta and Omicron BA.1 led to a significant increase in the proportion of multi-nucleated cells at 48 hpi, but the proportion of fused cells remained small (< 17%). Importantly, Omicron BA.2 did not significantly promote cell fusion, thus the latter could not account for the more severe phenotype we observed.

We next asked if the variants induced apoptosis and cell death differentially. Nuclear condensation was evaluated as a surrogate for apoptosis and appeared as bright and ‘condensed’ nuclei (Fig. 2E and Additional file 5, Fig. S2C). Omicron BA.2, but not the other variants, significantly increased the proportion of hiPSC-CMs with nuclear condensation compared to control, indicating an increased level of apoptosis at 48 hpi. A similar trend was observed with cleaved caspase 3 staining (Additional file 5, Fig. S2D). We also quantified the number of cells remaining after infection. Cell number was similar among all cultures at 24 hpi. However, BA.2 infection significantly reduced cell number by 37.5 ± 10.9% at 48 hpi, again suggesting pronounced cell death and detachment while the cell number was not noticeably altered by the other variants (Fig. 2F).

Overall, our results showed that all variants could induce cytopathic effects in hiPSC-CMs, with Omicron BA.2 inducing the most severe phenotype.

### Omicron BA.2 induces more severe myocardial damage than Delta in vivo

**Fig. 3:**
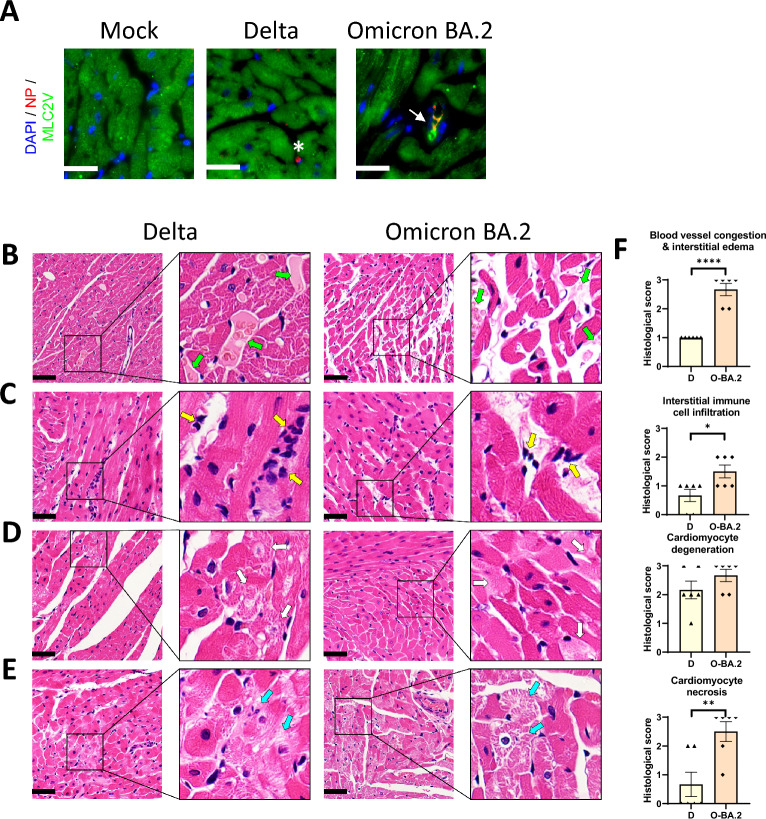
SARS-CoV-2 variants Delta and Omicron BA.2 infection induce cardiac damage in hamsters. Hamsters were inoculated intranasally with 10^4^ PFU SARS-CoV-2 Delta or Omicron BA.2 virus. At 2 days post infection (dpi), the hamsters were sacrificed. **A** Cryosections of the heart were stained with antibodies against the SARS-CoV-2 nucleocapsid protein (NP) in red, cardiac ventricular marker MLC2V in green, and DAPI nuclear staining in blue. Small clusters of NP^+^/MLC2V^+^ cells could be detected in Omicron BA.2 infected heart (arrow). NP^+^ cells could be detected in Delta-infected heart, but they are rarely positive for MLC2v (asterisk). **B**–**E** Paraffin sections of the heart were stained with H&E. Representative images of **B** myocardial blood vessel congestion and interstitial edema (green arrows), **C** interstitial immune cell infiltration (yellow arrows), **D** CM degeneration (white arrows). **E** CM necrosis (blue arrows). **F** Histological scores of pathological features scaled 0–3, where 0 indicates the absence of pathological changes. Data are presented as mean ± SEM, n = 3 for A, n = 6 for B-F . Statistical significance was calculated using Student’s t-test *p < 0.05, **p < 0.01, ****p < 0.0001. Scale bar **A** = 25 µM; **B**–**E** = 50 µM

Having demonstrated that different variants could differentially infect and damage CMs in vitro, we compared the effects of Delta, Omicron BA.1 and BA.2 infection on the heart in vivo*.* We utilised golden Syrian hamsters, which are susceptible to SARS-CoV-2 infection and are widely used as a non-lethal model to study the pathogenesis of COVID-19 [31–34]. Following intranasal inoculation, the presence of the viral NP could be observed in CMs (Fig. 3A), interstitial cells and endothelial cells (Additional file 6, Fig. S3A–E) in the hearts of infected hamsters by immunostaining at 2 dpi. To confirm cardiac infection, we co-stained our heart sections with antibodies against NP and MLC2V, a well-established cardiac ventricular marker (Fig. 3A). Small clusters of NP^+^ CMs could be detected in Omicron BA.2 infected heart. While the viral NP could be detected in the hearts of Delta- and Omicron BA.1 infected hamsters, cardiac infection was rare, and NP was mostly observed in non-CMs (Additional file 6, Fig. S3A–E).

Infected hamsters were then examined for signs of myocardial damage. Formalin-fixed and paraffin-embedded heart tissue sections were stained with Haematoxylin and Eosin (H&E) and examined under light microscope (Fig. 3B–E, Additional file 6, Fig. S3F). At 2 days after Omicron BA.2 or Delta infection, myocardium blood vessel congestion, interstitial edema and immune cell infiltrates as clusters of 3–5 cells were observed (Fig. 3B and C). Pericardium immune infiltrates mixed with fluid exudates were occasionally found in Omicron BA.2 infected hamsters. Immunostaining further confirmed the presence of CD45^+^ immune cells in Delta and Omicron BA.2 infected hearts (Additional file 6, Fig. S3G). Various degree of CM degenerative changes after Omicron BA.2 or Delta virus infection were found. The degenerated CMs were shown as loss of the cross-striation pattern and occasionally sarcoplasmic vacuolation which was more frequently found after Omicron BA. 2 infection (Fig. 3D). Necrotic changes of myocardium were observed in all 6 hamsters infected by Omicron BA.2, but it was less frequent after Delta infection at 2 dpi (2/6 hamsters) (Fig. 3E). Omicron BA.1 infected hamster showed histopathological changes of intermediate severity between Delta and Omicron BA.2 (Additional file 6, Fig. S3F). Since Omicron BA.2 and Delta represents the most extreme phenotypes in vitro (Fig. 2) and in vivo (Fig. 3B-E and Additional file 6, Fig. S3A–F), we focused our comparison on the two variants. The histopathological changes were scored based on how localised or widespread they were (score 0–3, high score indicates more widespread). Hamsters infected with Omicron BA.2 received a significantly higher score in CM necrosis, interstitial immune cell infiltration, blood vessel congestion and interstitial edema (Fig. 3F), as well as in the overall pathology compared to Delta (Additional file 6, Fig. S3H), indicating that Omicron BA.2 induced more widespread and severe myocardial damage than Delta in hamsters.

**Fig. 4:**
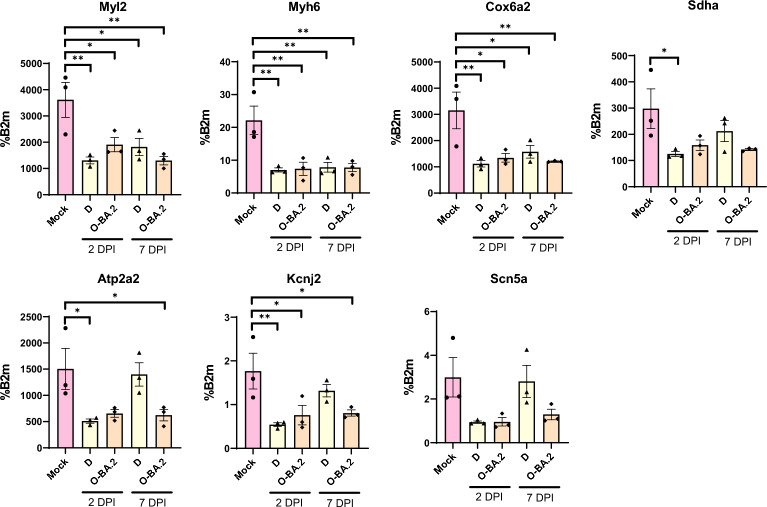
Gene expression analysis of hamster heart. RNA was extracted from hearts of hamsters infected with Delta or Omicron BA.2 at 2 or 7 dpi. The expression of genes important for cardiac function were measured by RT-qPCR, normalised to B2m expression. Data are presented as mean ± SEM, n = 3 biological replicates. Statistical analysis was performed using one-way ANOVA followed by Dunnett’s multiple comparisons test, relative to mock; *p < 0.05, **p < 0.01

To elucidate the pathogenesis of Omicron BA.2 and Delta in the heart, we next evaluated the expression of selected genes in Omicron BA.2 and Delta infected hearts at 2 dpi and 7 dpi by qRT-PCR (Fig. 4). Genes important for contraction (Myl2 and Myh6) and respiration (Cox6a2 and Sdha) were significantly repressed by both Omicron BA.2 and Delta, and these changes persisted till 7 dpi, when virus was no longer detectable. We also evaluated genes important for calcium (Ata2a2), potassium (Kcnj2) and sodium (Scn5a) transport. While they were similarly reduced by Omicron BA.2 and Delta at 2 dpi, these changes persisted in Omicron BA.2 infected hearts at 7 dpi, while there was a return to control levels in Delta samples.

Together, our histopathological evaluation and expression analysis indicates increased severity of Omicron BA.2 infection compared to Delta, which, particularly in the case of Omicron BA.2, persisted after viral clearance. Omicron BA.2 could infect CMs in vivo and might directly damage the heart. Conversely, cardiac infection by Delta was rare, thus the cardiac pathology observed, albeit weaker than that of Omicron BA.2, might arise via indirect damage.

### Omicron BA.2 does not induce more severe lung damage in vivo

We next evaluated the infectivity and pathology of Omicron BA.2 and Delta in the lung to test if the severe damage induced by Omicron BA.2 in the heart arise from more severe pulmonary injury. The plague assay revealed significantly lower viral titre in Omicron BA.2 infected lung than Delta (Additional file 7, Fig. S4A), consistent with previous reports in the respiratory system but contrary to our results in the heart. The lung sections of infected hamsters at 2 dpi showed peribronchiolar and perivascular infiltration, bronchiolar epithelium destruction with dead cell debris mixed with immune cells filled the lumine (Additional file 7, Fig. S4B). Alveolar wall capillary congestion and immune cell infiltration was moderate with patches areas of alveolar space infiltration. The histological damage mostly affected larger bronchi and surrounding tissue, alveoli in distal lung were less involved at 2 dpi. Signs of bronchiolitis, alveolitis and vasculitis were scored based on how localised or widespread they were (score 0–3, high score indicates more widespread) (Additional file 7, Fig. S4C). Hamsters infected with Omicron BA.2 received a similar score in bronchiolitis and alveolitis, and significantly lower score in vasculitis compared with Delta (Additional file 7, Fig. S4C). In summary, Omicron BA.2 induced a similar or slightly milder phenotype than Delta in the lungs, analogous to results reported in patients. Therefore, the more severe damage induced by Omicron BA.2 was not secondary to more severe infection or phenotype in the lungs.

### Omicron BA.2 induces expression changes indicative of compromised cardiac function

**Fig. 5:**
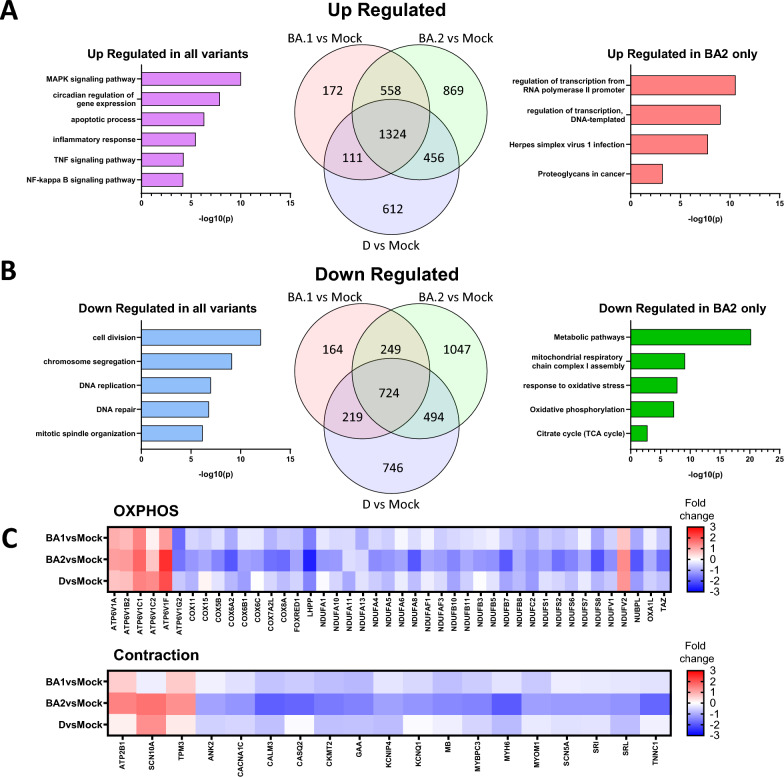
Transcriptomic profiling of hiPSC-CMs infected by SARS-CoV-2 variants. Human iPSC-CMs were infected with Delta (D), Omicron BA.1 (BA1) or BA.2 (BA2) for 48 h at MOI of 1, n = 3. Venn diagram showing the number of DEGs and their enrichment in different gene pathways commonly and specifically **A** up-, **B** down-regulated by the variants. **C** Heat map of selected genes associated with oxidative phosphorylation (OXPHOS) and contraction

**Fig. 6:**
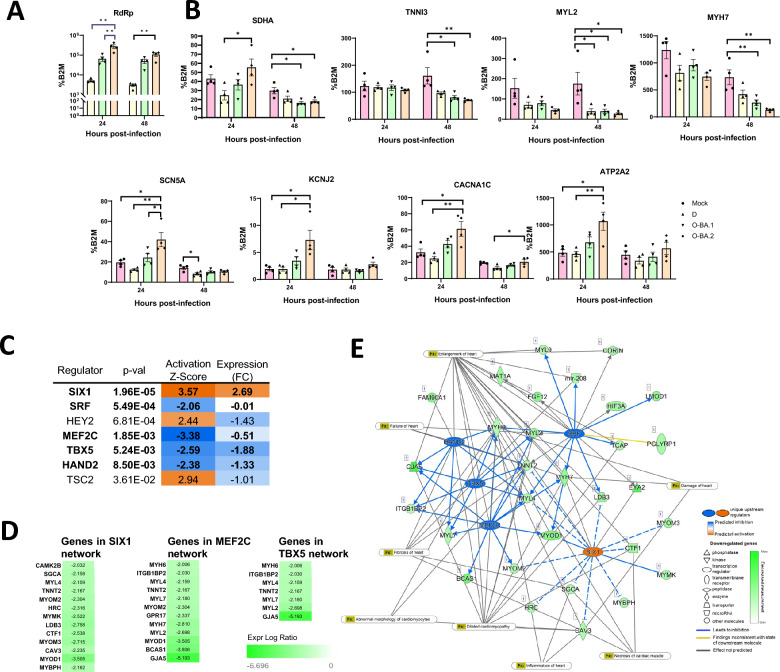
Delta, Omicron BA.1, and BA.2 induce distinct expression changes in hiPSC-CMs. Human iPSC-CMs were infected with Delta (D), Omicron BA.1 (O-BA.1) or BA.2 (O-BA.2) at MOI of 1. The expression of **A** RdRp, **B** genes important for cardiac function was measured by RT-qPCR, normalised to B2M expression. Data are mean ± SEM, n = 4 biological replicates. Statistical analysis was performed using one-way ANOVA followed by Tukey’s multiple comparisons test; *p < 0.05, **p < 0.01, ***p < 0.001, ****p < 0.0001. Differentially expressed genes in BA.2 group were examined by Ingenuity pathway analysis to predict upstream regulators. **C** Activation z-score indicates the activation (+) and inhibition (−) of the regulator, significance is indicated by the p-value of overlap. Expression is shown as log of fold change (FC). Regulators with consistent direction of activation score and expression change are shown in bold; **D** predicted downstream genes of selected regulators and **E** diagram of regulatory network, Fx: cardiac-related diseases and functions, Lines: direct (solid) and indirect (dotted) interactions

We have shown that the variants produce divergent cardiac effects in vitro and in vivo. To understand the molecular differences underlying these effects, we examined the global transcriptome of infected hiPSC-CMs by RNA-sequencing at 48 hpi (Figs. 5 and 6), and by RT-qPCR of selected genes at 24 and 48 hpi (Fig. 6A and B). RT-qPCR analysis revealed significantly higher levels of virally-encoded RdRp in hiPSC-CMs infected with Omicron BA.2 at 24 hpi, compared to other variants (Fig. 6A), in line with higher proportion of NP^+^ cells detected after Omicron BA.2 infection (Fig. 3A).

Comparison between infected and control hiPSC-CMs revealed (4687, Delta), (3521, Omicron BA.1), (5829, Omicron BA.2) differentially expressed genes (DEGs) mapped to the human transcriptome (> twofold). To identify molecular changes that are common or unique to the variants, we examined the intersection among the genes identified above. Venn diagram showed that 1324 and 724 transcripts were significantly up- and down-regulated by all three variants compared to control (Fig. 5A and B, Additional file 8, Table S3). Gene ontology and KEGG analyses showed that commonly upregulated genes were involved in signalling pathways important for inflammation and apoptosis. The most significantly upregulated genes involve those in the MAPK pathway [35]. Up-regulated genes also include components of the p53, TNF and NFκB pathways, which are all established regulators of the antiviral response. Down-regulated genes comprise components of cell cycle and DNA repair.

We next focused on Omicron BA.2 to understand why it induced a more severe phenotype in hiPSC-CMs. We examined genes that were uniquely and significantly altered by Omicron BA.2 and revealed downregulation of genes involved in mitochondrial function and energy production. In particular, a subset of genes involved in the mitochondrial electron transport chain, oxidative phosphorylation and tricarboxylic acid cycle were specifically suppressed by Omicron BA.2 (Fig. 5B and C) and this is consistent with the more severe mitochondrial phenotype induced by this variant (Fig. 2A and B). Heat map comparison further revealed greater suppression of contractile genes by Omicron BA.2 (Fig. 5C). We next evaluated genes known to be important for cardiac function by qPCR. We detected downregulation of SDHA, which plays a key role in oxidative phosphorylation in the mitochondria. (Fig. 6B). The levels of sarcomeric genes were more strongly reduced by Omicron BA.2 at 24 hpi (MYL2 and MYH7) and at 48 hpi (MYL2, MYH7 and TNNI3). The suppression of contractile genes by SARS-CoV-2 have been previously reported [16]. Our results showed that this is a common feature among variants while effects are most marked in Omicron BA.2. Lastly, we evaluated genes important for ion transport (sodium: SCN5A; potassium: KCNJ2; calcium: CACNA1C, ATP2A2). The expression of all four genes were significantly and most prominently altered by Omicron BA.2 at 24 hpi, suggestive of perturbed electrophysiology.

We used Ingenuity Pathway Analysis to identify upstream regulators based on prior knowledge of expected effects between transcriptional regulators and their target genes, to better understand why Omicron BA.2 more strongly downregulated genes important for cardiac function. Our analysis predicted that the transcription factors SIX1, TSC2 and HEY2 were uniquely activated in Omicron BA.2 infected cells, while SRF, MEF2C, TBX5, and HAND2 were uniquely suppressed (Fig. 6C). Specifically, they were predicted to significantly regulate gene expression in hiPSC-CMs infected by Omicron BA.2, but not other variants. Of these regulators, the expression of SIX1, MEF2C, TBX5, HAND2 and SRF were themselves altered in a manner consistent with its predicted activation/suppression in Omicron BA.2 infected hiPSC-CMs (Fig. 6C). The downstream targets of these regulators include genes important for contraction (TNNT2, MYL2) and Ca^2+^ handling (HRC). We then built an integrated network to illustrate co-operative regulation by these factors on downstream targets and how they are predicted to affect cardiac function and cause disease (Fig. 6D and E).

In summary, Omicron BA.2 induced expression changes associated with compromised contractile, mitochondrial and electrophysiological function, compared with other variants, and this may be related to dysregulation of a specific set of transcription factors.

### Omicron infects hiPSC-CMs via endocytosis

**Fig. 7:**
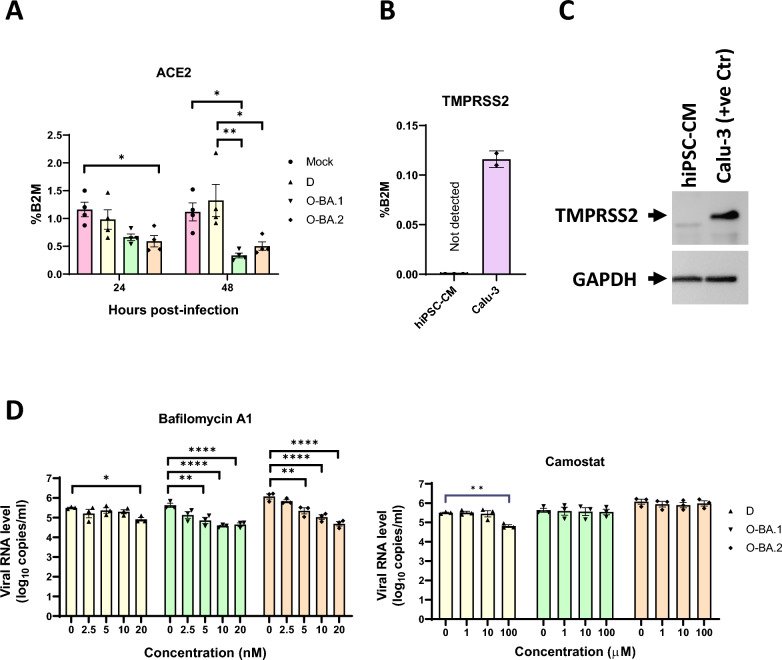
Omicron infection is mediated via endocytic pathway. Human iPSC-CMs were infected with Delta (D), Omicron BA.1 (O-BA.1) or BA.2 (O-BA.2) for 48 h at MOI of 1. **A** The level of ACE2 was measured by RT-qPCR, n = 4. **B** The level of TMPRSS2 mRNA in hiPSC-CMs (n = 3) and Calu-3 cells (positive control, n = 2) were measured by RT-qPCR; **C** TMPRSS2 protein was evaluated by western blot. **D** Human iPSC-CMs were infected with the three variants at an MOI of 1, treated with bafilomycin A1 or camostat at the indicated concentrations. Viral RNA levels of SARS-CoV-2 variants were determined by qPCR analysis. Data are presented as mean ± SEM, n refers to biological replicates. Statistical analysis was performed using **A** one-way ANOVA followed by Tukey’s multiple comparisons test, or **D** two-way ANOVA. D. Significance is shown relative to the control group (0 µM); *p < 0.05, **p < 0.01, ***p < 0.001, ****p < 0.0001

To elucidate the mechanism underlying differential infectivity of different variants in CMs, we investigated their viral entry pathways. SARS-CoV-2 has been shown to infect via cell membrane fusion and/or endocytosis. While host angiotensin-converting enzyme 2 (ACE2) is required for viral entry, cell membrane fusion depends on the cleavage of the viral spike protein by serine proteases such as TMPRSS2 to infect while the endocytosis does not require TMPRSS2. Thus, the expression of ACE2 and TMPRSS2 are important determinants of the tropism of SARS-CoV-2 variants. We examined the expression of ACE2 and TMPRSS2 in hiPSC-CMs to better understand the divergent abilities of the variants to infect these cells. RT-qPCR analysis revealed high levels of ACE2 in hiPSC-CMs. ACE2 is known to play a protective role in the cardiovascular system but its expression was significantly suppressed by Omicron BA.2 at 24 hpi and by both Omicron BA.1 and BA.2 at 48 hpi (Fig. 7A). TMPRSS2 mRNA and protein expression was abundant in Calu-3 human lung epithelial cells known to express this gene, but was below detection limit in hiPSC-CMs (Fig. 7B and C), consistent with previous reports showing the low/non-detectable expression of this gene in hPSC-CMs and adult CMs [36, 37].

In the respiratory system, Omicron BA.1 and BA.2 have been shown to infect via endocytosis in a TMPRSS2-independent manner, while Delta relies on TMPRSS2 to facilitate cell membrane fusion [6]. To test if this is also true in hiPSC-CMs, we assessed the abilities of inhibitors of endocytosis and cell membrane fusion to suppress infection (Fig. 7D, Additional file 9, Fig. S5). We evaluated viral replication in the presence of bafilomycin A1, which inhibits endocytosis. Omicron BA.1 and BA.2 both exhibited dose-dependent decreases in viral RNA level while Delta did not respond except at the highest concentration tested. E64d, which inhibits endocytosis by blocking cathepsins [5], induced similar effects: Omicron BA.2 experienced the largest decrease in viral RNA level. Conversely, camostat, which inhibits serine proteases such as TMPRSS2 [6], minimally affected viral RNA level except at the maximum dose where it reduced the copy number of Delta, consistent with the absence of TMPRSS2 in hiPSC-CMs. Our results demonstrate that Omicron BA.1 and BA.2, but not Delta, could infect hiPSC-CMs via endocytosis independently of TMPRSS2, which was absent in these cells.

## Discussion

SARS-CoV-2 variants are associated with divergent transmissibility and pathogenicity, and their tropism and cytopathic effects have been extensively investigated in the respiratory system [4–6, 8, 27]. However, less is known about the effect of variants in extrapulmonary tissues such as CMs. Using hiPSC-CMs and Golden Syrian hamsters as models, we demonstrate that different variants differentially infect and damage CMs. Specifically, Omicron BA.1 and BA.2 can efficiently infect CMs via the endocytic pathway independently of TMPRSS2, while Delta minimally infects these cells. Although Omicron BA.2 is commonly considered mild, Omicron BA.2 can induce a severe phenotype in vitro and in vivo. This is the first report to compare Delta and Omicron variants in CMs in vitro and in vivo, and demonstrate divergent effects.

Cardiac damage is a serious and potentially life-threatening complication of COVID-19. Elevated levels of biomarkers such as troponin, and myocardial dysfunction determined by echocardiography, are frequently detected in COVID-19 patients, and are associated with poor prognosis [38, 39]. How SARS-CoV-2 infection injures the heart remains elusive and direct infection of CMs has been proposed as one of the pathogenic mechanisms [16–22]. Many groups have employed hPSC-CM models to show that SARS-CoV-2 can directly infect CMs in vitro [16–21]. The presence of SARS-CoV-2 viral particles, RNA and protein, and signs of viral transcription (sense and antisense SARS-CoV-2 spike RNA) have been reported in CMs in endomyocardial biopsy and autopsy samples from COVID-19 patients, along with evidence of cardiac damage such as sarcomere rupture and cell death [20, 40–43]. Conversely, others reported the absence of CM infection in autopsy samples by single-cell RNA-sequencing and by dsRNA/protein analyses [21, 44]. Most studies involving clinical specimens use autopsy samples from patients long past the acute phase of infection. Importantly, most in vitro and clinical studies use viral isolates from early stages of the pandemic (Additional file 10, Table S4). To what extent direct CM infection contributes to cardiac damage in COVID-19 patients remains controversial. Here we show that different variants can divergently infect CMs and this may partially underlie the discrepant results reported. Omicron BA.1 and BA.2 can all efficiently infect CMs via endocytosis in an TMPRSS2-independent manner, while Delta was much less infectious. Our results are consistent with studies on the respiratory system, where Omicron can infect via endocytosis in the absence of TMPRSS2 while Delta relies on TMPRSS2 to infect via cell membrane fusion [4–6, 45]. Our in vitro results are supported by our hamster experiments, which demonstrate cardiac infection by Omicron BA.2 in vivo while Delta-infected CMs tended to be rare. Moreover, the more severe cardiac pathology induced by Omicron BA.2 occurred despite similar/milder pathology induced by this variant in the lung, showing that the former is not secondary to more severe respiratory disease. In summary, SARS-CoV-2 variants can damage the heart in different ways. The Delta variant, which causes severe respiratory illness, may predominantly damage the heart via hypoxemic stress and cytokine storm. Conversely or in addition, Omicron, particularly the BA.2 variant, may directly infect CMs and injure the heart.

Another key finding of this report is that Omicron BA.2 infection can induce severe phenotype. Our in vivo experiments demonstrated greater pathology in the heart of animals infected with Omicron BA.2 compared with Delta. In vitro, Omicron BA.2 was the only variant to significantly reduce redox potential (by > 64%) and induce cell detachment (by > 37%) at 48 hpi. We also detected more rapid and dramatic morphological deterioration in hiPSC-CMs following exposure to Omicron BA.2 compared to other variants. A recent manuscript showed increased viral replication and fusogenicity of Delta compared to Omicron BA.1 in hPSC-CMs and that Omicron BA.2 and BA.5 were more replicative than BA.1 [46]. Differences between this report and our data may relate to the status and age of hPSC-CMs used, which has been shown to affect the expression of entry factors such as TMPRSS2 in cells, which was not defined in this previous report. Our hPSC-CMs were cultured for > 40 days, and, similar to adult CMs, did not express TMPRSS2 [36, 37, 46]. Conversely, younger, and immature hPSC-CMs are positive for this transcript [46]. We also extended our analysis to hamster in vivo model to confirm the pathogenicity of Omicron BA2 in CMs.

Delta, Omicron BA.1 and BA.2 differ in their pathogenicity in respiratory cells and this has been attributed to the fusogenicity of these variants, mediated by TMPRSS2 [5, 30, 45, 47]. Contrary to these results, we observed only small increases in cell fusion in hiPSC-CMs infected by any variants compared to control, consistent with the absence of TMPRSS2 in hiPSC-CMs. Thus fusogenicity may not play a prominent role in the pathogenesis of SARS-CoV-2 induced CM damage and does not correlate with the more severe phenotype induced by Omicron BA.2. Instead, we seek to elucidate the mechanism by which Omicron BA.2 suppresses genes critical to cardiac function using bioinformatics analysis. Our analysis predicted SIX1, MEF2C, TBX5, HAND2 and SRF to be key regulators of the unique transcriptomic profile of Omicron BA.2 cardiac infection. SIX1 is an IFN stimulated transcription factor implicated in SARS-CoV-2 transcription [48] and here we predict that it is activated by Omicron BA.2. MEF2C, TBX5 and HAND2 are all transcription factors known to regulate expression of cardiac genes. For instance, TBX5 is critical for cardiac function [49] and its expression is dysregulated in the ventricular myocardium of heart failure patients [50]. Loss of TBX5 function in adult mice is associated with cardiac dysfunction, arrhythmias and sudden cardiac death [50]. Therefore its suppression by Omicron BA.2 may similarly damage CMs. Our analysis uncovered a previously unknown regulatory network that may contribute to altered cardiac gene expression and phenotype induced by Omicron BA.2.

Omicron is considered a ‘mild’ variant, with lower disease severity compared to previous variants such as Delta based on rate of hospitalisation and death [8, 51–53]. Delta is known to induce severe respiratory illness and inflammation, whose effects cannot be modelled using our in vitro CM model. Furthermore, we utilised a Golden Syrian Hamster model, which is a non-lethal COVID-19 model known to develop less severe disease than in humans, thus systemic cardiac damage induced by Delta might be underestimated. Due to these limitations, we do not claim that Omicron can induce more severe cardiac illness in patients. We do, however, conclude that Omicron BA.2 infection can damage CMs via different mechanisms compared with Delta, and proposed contributors to these effects in terms of dysregulation of transcription factors and different entry pathways. The Omicron is highly infectious and a sizable number of patients, particularly vulnerable patients such the elderly, the unvaccinated, and individuals with co-morbidities, are at risk of severe disease. A recent echocardiographic study showed that right ventricular function is impaired to a lower extent among Omicron patients compared with those infected with the wild-type variant and that this is potentially related to the attenuated pulmonary parenchymal and/or vascular disease in the former [54]. Yet, another recent study revealed broadly similar excess mortality attributed to acute myocardial infarction during the Omicron surge compared to early stages of the pandemic [55]. Indeed, an examination of heart transplant recipients demonstrated higher disease severity among Omicron compared to Delta patients [56]. Larger clinical studies are needed to compare the pathogenesis and sequelae of cardiac injury induced by different variants.

Human iPSC-CMs have been used in numerous publications to examine SARS-CoV-2 infection and its consequences [16–21, 57]. Human iPSC-CMs have been shown to be developmentally immature and resemble embryonic/fetal CMs, thus we subjected our cells to metabolic selection to promote more adult-like traits [24, 58–61]. In spite of this, it is possible that our hiPSC-CMs may not fully recapitulate the adult cardiac phenotype, and is a limitation of our in vitro study.

## Conclusions

In summary, we demonstrate that different variants can divergently infect and damage CMs. Specifically, we show that Omicron BA.2, which has infected large numbers of patients in much of the world, can infect CMs to cause cytopathic effects in vitro and in vivo despite milder pathology in the lung. Adult CMs have limited ability to regenerate, thus damage caused by viral infection can potentially cause irreparable harm to patients. Further clinical studies are warranted to study the pathogenic mechanisms of cardiac damage induced by different variants and to better monitor the long-term cardiovascular sequelae of COVID-19.

### Supplementary Information

Additional file 1. Supplementary methods.

Additional file 2: Table S1. Primers used for RT-qPCR.

Additional file 3: Table S2. Antibodies used for immunostaining and western blotting.

Additional file 4: Fig. S1. Delta, Omicron BA.1, and BA.2 differentially infect hiPSC-CMs. Human iPSC-CMs were exposed to Delta, Omicron BA.1, or BA.2, followed by immunostaining for NP. (A) Percentage of NP^+^ hiPSC-CMs infected at an MOI of 1 and harvested at 48 hpi, n = 4. (B) Percentage of NP^+^ hiPSC-CMs infected at an MOI of 0.1 and harvested at 24 or 48 hpi, n = 3. (C) Fluorescence images of the infected hiPSC-CMs, NP in Red, DAPI in blue. Data are presented as mean ± SEM, n refer to the number of biological replicates. Scale bar = 500 μm.

Additional file 5: Fig. S2. Omicron BA2 induces more severe damage in hiPSC-CMs. Human iPSC-CMs were infected with Delta (D), Omicron BA.1 (O-BA.1) or BA.2 (O-BA.2) for 48 h at an MOI of 1. (A) Representative images for scoring of mitochondrial fragmentation. Elongated, continuous mitochondria were scored as ‘Normal’, while punctate, discontinuous mitochondria are considered ‘Fragmented’. (B) Representative images of mock and infected hiPSC-CMs showing TOM20 immunostaining in yellow, DAPI nuclear staining in blue. (C) Representative images for scoring of nuclear condensation. Condensed nuclei are small, brightly stained, with irregular shapes. Multi-nucleated cells were defined as cells which contained more than one nucleus in close proximity of each other. (D) The percentage of hiPSC-CMs with cleaved caspase 3 staining was measured, n = 3. Scale bar A and B = 10 μm, C = 25 μm.

Additional file 6: Fig. S3. Infection of SARS-CoV-2 Delta and Omicron variants (BA.1 and BA.2 variants) in hamster heart. Groups of hamsters were inoculated intranasally with 10^4^ PFU SARS-CoV-2 Delta, Omicron BA.1, BA.2 virus, or mock control. The hamsters were sacrificed at 2 dpi. Immunohistochemistry staining of SARS-CoV-2 nucleoprotein (NP) on heart sections were performed; positive cells were stained in brown color. Representative images of NP staining (A, C) Interstitial cells, (B, D) endothelial cells and (E) Mock, scale bar = 50 µm. (F) Representative images of H&E stained sections of Omicron BA.1 infected heart showing mild histopathological changes including perivascular immune cell accumulation (i, arrows), degenerated cardiomyocytes (ii, arrows) and a few foci of cardiomyocytes necrotic changes (iii, arrows). (G) Cryosections of the heart were stained with antibodies against CD45 in red, and DAPI nuclear staining in blue, showing sign of interstitial immune cell infiltration, scale bar = 25 µm. (H) Sum of histological scores of heart sections of all four pathological features, with maximal possible score of 12. Data are presented as mean ± SEM. n = 6 biological replicates for A, B, E, G and H; n = 3 for C, D, F. Statistical significance was calculated using Student’s t-test **p < 0.01, ***p < 0.001.

Additional file 7: Fig. S4. Infection of SARS-CoV-2 Delta and Omicron BA.2 variants in hamster lung. Groups of hamsters were inoculated intranasally with 10^4^ PFU SARS-CoV-2 Delta, Omicron BA.2 virus, or mock control. The hamsters were sacrificed at 2 dpi; lung was fixed in formalin, processed to paraffin sections. (A) The viral titre in the lungs was determined using the plague assay. (n = 3) (B) H&E staining on hamster lung tissues were performed, representative images were shown. Mock control hamsters showed normal histological structures. Omicron BA.2 infected hamster lung showed bronchiolar epithelial cell damage with cell debris filled the lumen. Immune cell infiltration in the area surrounding the bronchiole and the vasculatures. Similar histological changes were also shown in Delta virus infected lung. Scale bar = 200 µm. (C) Histological scores of pathological features in the lungs, scaled 0–3, where 0 indicates the absence of pathological changes. Sum of histological scores of all three pathological features, with maximal possible score of 9. Data are presented as mean ± SEM, n = 6 biological replicates unless otherwise indicated. Statistical significance was calculated using Student’s t-test **p < 0.01, ***p < 0.001.

Additional file 8: Table S3. A. Biological processes and pathways significantly enriched among DEGs commonly up- or down-regulated by all three variants in hiPSC-CMs. Table S3B. Biological processes and pathways significantly enriched among DEGs uniquely altered by Omicron BA.2.

Additional file 9: Fig. S5. SARS-CoV-2 infection is inhibited by endocytosis inhibitor. Human iPSC-CMs were infected with Delta (D), Omicron BA.1 (O-BA.1) or BA.2 (O-BA.2) for 48 h at an MOI of 1, treated with E64d at the indicated concentrations. Viral RNA levels of SARS-CoV-2 variants were determined by qPCR analysis. Data are presented as mean ± SEM, n = 4 biological replicates. Significance is calculated in respect to the control group (0 µM); **p < 0.01, ***p < 0.001, ****p < 0.0001.

Additional file 10: Table S4. In vitro studies of SARS-CoV-2 using hPSC-CM models.

## Data Availability

The main data supporting the findings of this study are available within the paper. Further information and requests for resources and reagents should be directed to the corresponding author.

## Abbreviations

ACE2: Angiotensin-converting enzyme 2
CMs: Cardiomyocytes
COVID-19: Coronavirus disease 2019
DEGs: Differentially expressed genes
dpi: Days post infection
eGFP: Enhanced green fluorescent protein
SARS-CoV-2: Severe acute respiratory syndrome coronavirus 2
H&E: Haematoxylin and Eosin
hpi: Hours post infection
hiPSC-: Human induced pluripotent stem cell-derived
hiPSC-CMs: Human induced pluripotent stem cells-derived cardiomyocytes
NP: Nucleocapsid protein
SEM: Standard error of the mean
TMPRSS2: Transmembrane serine protease 2

## References

[1] To KK, Sridhar S, Chiu KH, Hung DL, Li X, Hung IF, Tam AR, Chung TW, Chan JF, Zhang AJ, et al. Lessons learned 1 year after SARS-CoV-2 emergence leading to COVID-19 pandemic. Emerg Microbes Infect. 2021;10(1):507–35. 33666147 10.1080/22221751.2021.1898291 PMC8006950

[2] Viana R, Moyo S, Amoako DG, Tegally H, Scheepers C, Althaus CL, Anyaneji UJ, Bester PA, Boni MF, Chand M, et al. Rapid epidemic expansion of the SARS-CoV-2 Omicron variant in southern Africa. Nature. 2022;603(7902):679–86. 35042229 10.1038/s41586-022-04411-y PMC8942855

[3] Cheng VCC, Ip JD, Chu AWH, Tam AR, Chan WM, Abdullah SMU, Chan BPC, Wong SC, Kwan MYW, Chua GT, et al. Rapid spread of severe acute respiratory syndrome coronavirus 2 (SARS-CoV-2) Omicron subvariant BA.2 in a single-source community outbreak. Clin Infect Dis. 2022;75(1):e44–9. 35271728 10.1093/cid/ciac203 PMC8992238

[4] Hui KPY, Ho JCW, Cheung MC, Ng KC, Ching RHH, Lai KL, Kam TT, Gu H, Sit KY, Hsin MKY, et al. SARS-CoV-2 Omicron variant replication in human bronchus and lung ex vivo. Nature. 2022;603(7902):715–20. 35104836 10.1038/s41586-022-04479-6

[5] Meng B, Abdullahi A, Ferreira I, Goonawardane N, Saito A, Kimura I, Yamasoba D, Gerber PP, Fatihi S, Rathore S, et al. Altered TMPRSS2 usage by SARS-CoV-2 Omicron impacts infectivity and fusogenicity. Nature. 2022;603(7902):706–14. 35104837 10.1038/s41586-022-04474-x PMC8942856

[6] Zhao H, Lu L, Peng Z, Chen LL, Meng X, Zhang C, Ip JD, Chan WM, Chu AW, Chan KH, et al. SARS-CoV-2 Omicron variant shows less efficient replication and fusion activity when compared with Delta variant in TMPRSS2-expressed cells. Emerg Microbes Infect. 2022;11(1):277–83. 34951565 10.1080/22221751.2021.2023329 PMC8774049

[7] Liu L, Iketani S, Guo Y, Chan JF, Wang M, Liu L, Luo Y, Chu H, Huang Y, Nair MS, et al. Striking antibody evasion manifested by the Omicron variant of SARS-CoV-2. Nature. 2022;602(7898):676–81. 35016198 10.1038/s41586-021-04388-0

[8] Shuai H, Chan JF, Hu B, Chai Y, Yuen TT, Yin F, Huang X, Yoon C, Hu JC, Liu H, et al. Attenuated replication and pathogenicity of SARS-CoV-2 B.1.1.529 Omicron. Nature. 2022;603(7902):693–9. 35062016 10.1038/s41586-022-04442-5

[9] Hoffmann M, Kleine-Weber H, Schroeder S, Kruger N, Herrler T, Erichsen S, Schiergens TS, Herrler G, Wu NH, Nitsche A, et al. SARS-CoV-2 cell entry depends on ACE2 and TMPRSS2 and is blocked by a clinically proven protease inhibitor. Cell. 2020;181(2):271-280.e278. 32142651 10.1016/j.cell.2020.02.052 PMC7102627

[10] Shi S, Qin M, Shen B, Cai Y, Liu T, Yang F, Gong W, Liu X, Liang J, Zhao Q, et al. Association of cardiac injury with mortality in hospitalized patients with COVID-19 in Wuhan, China. JAMA Cardiol. 2020;5(7):802–10. 32211816 10.1001/jamacardio.2020.0950 PMC7097841

[11] Task Force for the management of C-otESoC, Baigent C, Windecker S, Andreini D, Arbelo E, Barbato E, Bartorelli AL, Baumbach A, Behr ER, Berti S, et al. European Society of Cardiology guidance for the diagnosis and management of cardiovascular disease during the COVID-19 pandemic: part 1-epidemiology, pathophysiology, and diagnosis. Cardiovasc Res. 2022;118(6):1385–412. 34864874 10.1093/cvr/cvab342 PMC8690255

[12] Xie Y, Xu E, Bowe B, Al-Aly Z. Long-term cardiovascular outcomes of COVID-19. Nat Med. 2022;28(3):583–90. 35132265 10.1038/s41591-022-01689-3 PMC8938267

[13] Nishiga M, Wang DW, Han Y, Lewis DB, Wu JC. COVID-19 and cardiovascular disease: from basic mechanisms to clinical perspectives. Nat Rev Cardiol. 2020;17(9):543–58. 32690910 10.1038/s41569-020-0413-9 PMC7370876

[14] Magadum A, Kishore R. Cardiovascular manifestations of COVID-19 infection. Cells. 2020;9(11):2508. 33228225 10.3390/cells9112508 PMC7699571

[15] Gopal R, Marinelli MA, Alcorn JF. Immune mechanisms in cardiovascular diseases associated with viral infection. Front Immunol. 2020;11: 570681. 33193350 10.3389/fimmu.2020.570681 PMC7642610

[16] Sharma A, Garcia G Jr, Wang Y, Plummer JT, Morizono K, Arumugaswami V, Svendsen CN. Human iPSC-derived cardiomyocytes are susceptible to SARS-CoV-2 infection. Cell Rep Med. 2020;1(4): 100052. 32835305 10.1016/j.xcrm.2020.100052 PMC7323681

[17] Yang L, Han Y, Nilsson-Payant BE, Gupta V, Wang P, Duan X, Tang X, Zhu J, Zhao Z, Jaffre F, et al. A human pluripotent stem cell-based platform to study SARS-CoV-2 tropism and model virus infection in human cells and organoids. Cell Stem Cell. 2020;27(1):125-136.e127. 32579880 10.1016/j.stem.2020.06.015 PMC7303620

[18] Wichmann D, Sperhake JP, Lutgehetmann M, Steurer S, Edler C, Heinemann A, Heinrich F, Mushumba H, Kniep I, Schroder AS, et al. Autopsy findings and venous thromboembolism in patients with COVID-19: a prospective cohort study. Ann Intern Med. 2020;173(4):268–77. 32374815 10.7326/M20-2003 PMC7240772

[19] Bose RJC, McCarthy JR. Direct SARS-CoV-2 infection of the heart potentiates the cardiovascular sequelae of COVID-19. Drug Discov Today. 2020;25(9):1559–60. 32592868 10.1016/j.drudis.2020.06.021 PMC7313487

[20] Bojkova D, Wagner JUG, Shumliakivska M, Aslan GS, Saleem U, Hansen A, Luxan G, Gunther S, Pham MD, Krishnan J, et al. SARS-CoV-2 infects and induces cytotoxic effects in human cardiomyocytes. Cardiovasc Res. 2020;116(14):2207–15. 32966582 10.1093/cvr/cvaa267 PMC7543363

[21] Perez-Bermejo JA, Kang S, Rockwood SJ, Simoneau CR, Joy DA, Silva AC, Ramadoss GN, Flanigan WR, Fozouni P, Li H, et al. SARS-CoV-2 infection of human iPSC-derived cardiac cells reflects cytopathic features in hearts of patients with COVID-19. Sci Transl Med. 2021;13(590): eabf7872. 33723017 10.1126/scitranslmed.abf7872 PMC8128284

[22] Yang Y, Wei Z, Xiong C, Qian H. Direct mechanisms of SARS-CoV-2-induced cardiomyocyte damage: an update. Virol J. 2022;19(1):108. 35752810 10.1186/s12985-022-01833-y PMC9233758

[23] Kwon Y, Nukala SB, Srivastava S, Miyamoto H, Ismail NI, Jousma J, Rehman J, Ong SB, Lee WH, Ong SG. Detection of viral RNA fragments in human iPSC cardiomyocytes following treatment with extracellular vesicles from SARS-CoV-2 coding sequence overexpressing lung epithelial cells. Stem Cell Res Ther. 2020;11(1):514. 33256833 10.1186/s13287-020-02033-7 PMC7703503

[24] Kwok M, Lee C, Li HS, Deng R, Tsoi C, Ding Q, Tsang SY, Leung KT, Yan BP, Poon EN. Remdesivir induces persistent mitochondrial and structural damage in human induced pluripotent stem cell-derived cardiomyocytes. Cardiovasc Res. 2022;118(12):2652–64. 34609482 10.1093/cvr/cvab311 PMC8500104

[25] Waas M, Weerasekera R, Kropp EM, Romero-Tejeda M, Poon EN, Boheler KR, Burridge PW, Gundry RL. Are these cardiomyocytes? Protocol development reveals impact of sample preparation on the accuracy of identifying cardiomyocytes by flow cytometry. Stem Cell Rep. 2019;12(2):395–410. 10.1016/j.stemcr.2018.12.016 PMC6373208 30686762

[26] Tsoi C, Deng R, Kwok M, Yan B, Lee C, Li HS, Ma CHY, Luo R, Leung KT, Chan GC, et al. Temporal control of the WNT signaling pathway during cardiac differentiation impacts upon the maturation state of human pluripotent stem cell derived cardiomyocytes. Front Mol Biosci. 2022;9: 714008. 35402504 10.3389/fmolb.2022.714008 PMC8987729

[27] Yuan S, Ye ZW, Liang R, Tang K, Zhang AJ, Lu G, Ong CP, Man Poon VK, Chan CC, Mok BW, et al. Pathogenicity, transmissibility, and fitness of SARS-CoV-2 Omicron in Syrian hamsters. Science. 2022;377(6604):428–33. 35737809 10.1126/science.abn8939

[28] Ramachandran K, Maity S, Muthukumar AR, Kandala S, Tomar D, Abd El-Aziz TM, Allen C, Sun Y, Venkatesan M, Madaris TR, et al. SARS-CoV-2 infection enhances mitochondrial PTP complex activity to perturb cardiac energetics. iScience. 2022;25(1): 103722. 35005527 10.1016/j.isci.2021.103722 PMC8720045

[29] Mehrzadi S, Karimi MY, Fatemi A, Reiter RJ, Hosseinzadeh A. SARS-CoV-2 and other coronaviruses negatively influence mitochondrial quality control: beneficial effects of melatonin. Pharmacol Ther. 2021;224: 107825. 33662449 10.1016/j.pharmthera.2021.107825 PMC7919585

[30] Saito A, Irie T, Suzuki R, Maemura T, Nasser H, Uriu K, Kosugi Y, Shirakawa K, Sadamasu K, Kimura I, et al. Enhanced fusogenicity and pathogenicity of SARS-CoV-2 Delta P681R mutation. Nature. 2022;602(7896):300–6. 34823256 10.1038/s41586-021-04266-9 PMC8828475

[31] Rizvi ZA, Dalal R, Sadhu S, Binayke A, Dandotiya J, Kumar Y, Shrivastava T, Gupta SK, Aggarwal S, Tripathy MR, et al. Golden Syrian hamster as a model to study cardiovascular complications associated with SARS-CoV-2 infection. Elife. 2022;11: e73522. 35014610 10.7554/eLife.73522 PMC8794466

[32] Chan JF, Zhang AJ, Yuan S, Poon VK, Chan CC, Lee AC, Chan WM, Fan Z, Tsoi HW, Wen L, et al. Simulation of the clinical and pathological manifestations of coronavirus disease 2019 (COVID-19) in a golden Syrian hamster model: implications for disease pathogenesis and transmissibility. Clin Infect Dis. 2020;71(9):2428–46. 32215622 10.1093/cid/ciaa325 PMC7184405

[33] Sia SF, Yan LM, Chin AWH, Fung K, Choy KT, Wong AYL, Kaewpreedee P, Perera R, Poon LLM, Nicholls JM, et al. Pathogenesis and transmission of SARS-CoV-2 in golden hamsters. Nature. 2020;583(7818):834–8. 32408338 10.1038/s41586-020-2342-5 PMC7394720

[34] Rosenke K, Meade-White K, Letko M, Clancy C, Hansen F, Liu Y, Okumura A, Tang-Huau TL, Li R, Saturday G, et al. Defining the Syrian hamster as a highly susceptible preclinical model for SARS-CoV-2 infection. Emerg Microbes Infect. 2020;9(1):2673–84. 33251966 10.1080/22221751.2020.1858177 PMC7782266

[35] Weckbach LT, Schweizer L, Kraechan A, Bieber S, Ishikawa-Ankerhold H, Hausleiter J, Massberg S, Straub T, Klingel K, Grabmaier U, et al. Association of complement and MAPK activation with SARS-CoV-2-associated myocardial inflammation. JAMA Cardiol. 2022;7(3):286–97. 34910083 10.1001/jamacardio.2021.5133 PMC8674808

[36] Navaratnarajah CK, Pease DR, Halfmann PJ, Taye B, Barkhymer A, Howell KG, Charlesworth JE, Christensen TA, Kawaoka Y, Cattaneo R, et al. Highly efficient SARS-CoV-2 infection of human cardiomyocytes: spike protein-mediated cell fusion and its inhibition. J Virol. 2021;95(24): e0136821. 34613786 10.1128/JVI.01368-21 PMC8610601

[37] Litvinukova M, Talavera-Lopez C, Maatz H, Reichart D, Worth CL, Lindberg EL, Kanda M, Polanski K, Heinig M, Lee M, et al. Cells of the adult human heart. Nature. 2020;588(7838):466–72. 32971526 10.1038/s41586-020-2797-4 PMC7681775

[38] Madjid M, Safavi-Naeini P, Solomon SD, Vardeny O. Potential effects of coronaviruses on the cardiovascular system: a review. JAMA Cardiol. 2020;5(7):831–40. 32219363 10.1001/jamacardio.2020.1286

[39] Bansal M. Cardiovascular disease and COVID-19. Diabetes Metab Syndr. 2020;14(3):247–50. 32247212 10.1016/j.dsx.2020.03.013 PMC7102662

[40] Bulfamante GP, Perrucci GL, Falleni M, Sommariva E, Tosi D, Martinelli C, Songia P, Poggio P, Carugo S, Pompilio G. Evidence of SARS-CoV-2 transcriptional activity in cardiomyocytes of COVID-19 patients without clinical signs of cardiac involvement. Biomedicines. 2020;8(12):626. 33352880 10.3390/biomedicines8120626 PMC7767122

[41] Nakamura Y, Katano H, Nakajima N, Sato Y, Suzuki T, Sekizuka T, Kuroda M, Izutani Y, Morimoto S, Maruyama J, et al. SARS-CoV-2 is localized in cardiomyocytes: a postmortem biopsy case. Int J Infect Dis. 2021;111:43–6. 34384897 10.1016/j.ijid.2021.08.015 PMC8351278

[42] Bailey AL, Dmytrenko O, Greenberg L, Bredemeyer AL, Ma P, Liu J, Penna V, Winkler ES, Sviben S, Brooks E, et al. SARS-CoV-2 infects human engineered heart tissues and models COVID-19 myocarditis. JACC Basic Transl Sci. 2021;6(4):331–45. 33681537 10.1016/j.jacbts.2021.01.002 PMC7909907

[43] Albert CL, Carmona-Rubio AE, Weiss AJ, Procop GG, Starling RC, Rodriguez ER. The enemy within: sudden-onset reversible cardiogenic shock with biopsy-proven cardiac myocyte infection by severe acute respiratory syndrome coronavirus 2. Circulation. 2020;142(19):1865–70. 32997947 10.1161/CIRCULATIONAHA.120.050097

[44] Brauninger H, Stoffers B, Fitzek ADE, Meissner K, Aleshcheva G, Schweizer M, Weimann J, Rotter B, Warnke S, Edler C, et al. Cardiac SARS-CoV-2 infection is associated with pro-inflammatory transcriptomic alterations within the heart. Cardiovasc Res. 2022;118(2):542–55. 34647998 10.1093/cvr/cvab322 PMC8803085

[45] Peacock TP, Brown JC, Zhou J, Thakur N, Newman J, Kugathasan R, Sukhova K, Kaforou M, Bailey D, Barclay WS. The SARS-CoV-2 variant, Omicron, shows rapid replication in human primary nasal epithelial cultures and efficiently uses the endosomal route of entry. bioRxiv. 2022. 10.1101/2021.12.31.474653. 10.1101/2021.12.31.474653

[46] Nchioua R, Diofano F, Noettger S, von Maltitz P, Stenger S, Zech F, Munch J, Sparrer KMJ, Just S, Kirchhoff F. Strong attenuation of SARS-CoV-2 Omicron BA.1 and increased replication of the BA.5 subvariant in human cardiomyocytes. Signal Transduct Target Ther. 2022;7(1):395. 36566244 10.1038/s41392-022-01256-9 PMC9789943

[47] Yamasoba D, Kimura I, Nasser H, Morioka Y, Nao N, Ito J, Uriu K, Tsuda M, Zahradnik J, Shirakawa K, et al. Virological characteristics of SARS-CoV-2 BA.2 variant. BioRxiv. 2022. 10.1101/2022.02.14.480335. 10.1101/2022.02.14.480335

[48] di Bari I, Franzin R, Picerno A, Stasi A, Cimmarusti MT, Di Chiano M, Curci C, Pontrelli P, Chironna M, Castellano G, et al. Severe acute respiratory syndrome coronavirus 2 may exploit human transcription factors involved in retinoic acid and interferon-mediated response: a hypothesis supported by an in silico analysis. New Microbes New Infect. 2021;41: 100853. 33680474 10.1016/j.nmni.2021.100853 PMC7912353

[49] Basson CT, Bachinsky DR, Lin RC, Levi T, Elkins JA, Soults J, Grayzel D, Kroumpouzou E, Traill TA, Leblanc-Straceski J, et al. Mutations in human TBX5 [corrected] cause limb and cardiac malformation in Holt-Oram syndrome. Nat Genet. 1997;15(1):30–5. 8988165 10.1038/ng0197-30

[50] Rathjens FS, Blenkle A, Iyer LM, Renger A, Syeda F, Noack C, Jungmann A, Dewenter M, Toischer K, El-Armouche A, et al. Preclinical evidence for the therapeutic value of TBX5 normalization in arrhythmia control. Cardiovasc Res. 2021;117(8):1908–22. 32777030 10.1093/cvr/cvaa239 PMC8262635

[51] Sigal A, Milo R, Jassat W. Estimating disease severity of Omicron and Delta SARS-CoV-2 infections. Nat Rev Immunol. 2022;22(5):267–9. 35414124 10.1038/s41577-022-00720-5 PMC9002222

[52] Paredes MI, Lunn SM, Famulare M, Frisbie LA, Painter I, Burstein R, Roychoudhury P, Xie H, Mohamed Bakhash SA, Perez R, et al. Associations between severe acute respiratory syndrome coronavirus 2 (SARS-CoV-2) variants and risk of coronavirus disease 2019 (COVID-19) hospitalization among confirmed cases in washington state: a retrospective cohort study. Clin Infect Dis. 2022;75(1):e536–44. 35412591 10.1093/cid/ciac279 PMC9047245

[53] Fall A, Eldesouki RE, Sachithanandham J, Morris CP, Norton JM, Gaston DC, Forman M, Abdullah O, Gallagher N, Li M, et al. The displacement of the SARS-CoV-2 variant Delta with Omicron: an investigation of hospital admissions and upper respiratory viral loads. EBioMedicine. 2022;79: 104008. 35460989 10.1016/j.ebiom.2022.104008 PMC9020587

[54] Ghantous E, Shetrit A, Hochstadt A, Banai A, Lupu L, Levi E, Szekely Y, Schellekes N, Jacoby T, Zahler D, et al. Cardiologic manifestations in omicron-type versus wild-type COVID-19: a systematic echocardiographic study. J Am Heart Assoc. 2023;12(3): e027188. 36695308 10.1161/JAHA.122.027188 PMC9973649

[55] Yeo YH, Wang M, He X, Lv F, Zhang Y, Zu J, Li M, Jiao Y, Ebinger JE, Patel JK, et al. Excess risk for acute myocardial infarction mortality during the COVID-19 pandemic. J Med Virol. 2023;95(1): e28187. 36176195 10.1002/jmv.28187 PMC9839603

[56] Hazan F, Verdonk C, Coutance G, Ferré VM, Marot S, Melo VDD, Legeai C, Lebreton G, Para M, Varnous S, et al. Severity of SARS-CoV-2 Omicron variant infection in heart transplant recipients. J Heart Lung Transplant. 2023;42(5):558–61. 36822931 10.1016/j.healun.2023.01.012 PMC9890932

[57] Rockwood SJ, Arzt M, Sharma A. Modeling cardiac SARS-CoV-2 infection with human pluripotent stem cells. Curr Cardiol Rep. 2022;24(12):2121–9. 36272051 10.1007/s11886-022-01813-2 PMC9589554

[58] Poon EN, Luo XL, Webb SE, Yan B, Zhao R, Wu SCM, Yang Y, Zhang P, Bai H, Shao J, et al. The cell surface marker CD36 selectively identifies matured, mitochondria-rich hPSC-cardiomyocytes. Cell Res. 2020;30(7):626–9. 32157205 10.1038/s41422-020-0292-y PMC7343859

[59] Boheler KR, Poon EN. Cell surface markers for immunophenotyping human pluripotent stem cell-derived cardiomyocytes. Pflugers Arch. 2021;473(7):1023–39. 33928456 10.1007/s00424-021-02549-8

[60] Guo Y, Pu WT. Cardiomyocyte maturation: new phase in development. Circ Res. 2020;126(8):1086–106. 32271675 10.1161/CIRCRESAHA.119.315862 PMC7199445

[61] Yang X, Rodriguez ML, Leonard A, Sun L, Fischer KA, Wang Y, Ritterhoff J, Zhao L, Kolwicz SC Jr, Pabon L, et al. Fatty acids enhance the maturation of cardiomyocytes derived from human pluripotent stem cells. Stem Cell Rep. 2019;13(4):657–68. 10.1016/j.stemcr.2019.08.013 PMC6829750 31564645

