# Supplementary material for: SARS-CoV-2 variants divergently infect and damage cardiomyocytes in vitro and in vivo
Source: Cell Biosci. 2024 Aug 2;14:101. doi: 10.1186/s13578-024-01280-y (PMC11297708; doi:10.1186/s13578-024-01280-y)
Supplement: Supplementary file 1 — Additional file 1. Supplementary methods. [file 13578_2024_1280_MOESM1_ESM.pdf]

Supplementary Methods

Materials
Reagents, unless otherwise specified, were purchased from Sigma-Aldrich.

Cell Culture
Vero E6 cells expressing the TMPRSS2 (VeroE6/TMPRSS2) were obtained from JCRB Cell Bank (JCRB1819) and were maintained in DMEM, 10% FBS, 1% P/S and 1mg/ml of geneticin. All cell lines were maintained at 37 °C, 5% CO_2.

Viral culture
The SARS-CoV-2 variants, Delta (GISAID: EPI_ISL_3221329), Omicron BA.1 (GenBank: OM212472) and Omicron BA.2 (GISAID: *EPI_ISL_9845731*) were isolated from specimens obtained from three laboratory-confirmed COVID-19 patients. Vero E6/TMPRSS2 cells were used for viral propagation. All experiments involving SARS-CoV-2 viruses were conducted in a Biosafety Level-3 laboratory. Viral stocks were prepared and divided into aliquots. Viral titres were determined by plaque assay using Vero E6/TMPRSS2 cells. In brief, confluent monolayer of Vero E6/TMPRSS2 cells were washed with PBS, then serial dilutions of inoculum, diluted in complete medium (DMEM, 10% FBS, 1% P/S) were overlayed onto cells for 1 h at 37 °C. Inoculum was then removed and replaced with 1% w/v agarose in complete medium. Plates were incubated for 2 or 3d at 37 °C before fixation with 10% formalin. Overlay was removed 24 hr after fixation and cells were stained for 1 min at room temperature in crystal violet solution.

Gene expression analysis by RT-qPCR
The transcription levels of target genes were evaluated with the use of GoTaq™ qPCR Master Mix (Promega, Madison, WI) and QuantStudio 12K Flex (ThermoFisher Scientific, Waltham, MA). The data was normalised to B2M and was compared with the mock control or the untreated control. A detailed list of primer sequences for RT-qPCR is shown in additional file 2, Table S1.

Human iPSC culture and cardiac differentiation
Human iPSC line AICS-0060-027 (Allen Cell Collection) was used in our experiments. It is a derivative of the WTC-11 parental line with a mono-allelic mEGFP-tagged MYL2 modification. Undifferentiated AICS-0060-027 hiPSCs were maintained on Matrigel-coated surface (Corning Life Science, Tewksbury, MA) with mTeSR medium plus supplements (Stem Cell Technologies, Vancouver, Canada) at 37ºC with 5% CO_2. Cardiac differentiation was performed as described[1]. Undifferentiated hiPSCs were plated on individual wells of 6-well plate 3 days prior to differentiation. For cardiac differentiation, the mTeSR plus medium was changed to RPMI 1640/B27-insulin (B27 supplement without insulin) (ThermoFisher Scientific, Waltham, MA). On day 0 to day 2, 7.5 µM of CHIR99021 (Cayman Chemical, Ann Arbor, MI) was added to the cells. This was followed by incubation with RPMI 1640/B27-insulin medium and 7.5 µM of IWP-2 (APExBIO, Boston, MA) and 5 µM of IWR-1-endo (Cayman Chemical, Ann Arbor, MI) from day 2 to day 4. Cells were kept in RPMI 1640/B27-insulin from day 4 to day 8. The culture was maintained in RPMI 1640/B27+insulin (B27 supplement with insulin) (ThermoFisher Scientific, Waltham, MA) after day 8, at which point the cultures usually started to contract spontaneously, and media was replenished every 4 days. Metabolic selection of CMs was performed by changing the culture medium to RPMI 1640 (without glucose)/B27 supplemented with Linoleic acid-albumin, Oleic acid-albumin and triiodothyronine on day 18 to day 21. The hiPSC-CMs were maintained for at least 40 days

before use in experiments and >90% cells were positive for cardiac troponin T by flow cytometry[2, 3].

Infection of hiPSC-CMs
Human iPSC-CMs were infected at a multiplicity of infection (MOI) of 1, unless otherwise stated, for phenotypic analysis and RT-qPCR experiments. Human iPSC-CMs were fixed with 4% paraformaldehyde or lysed with Trizol reagent (ThermoFisher Scientific, Waltham, MA) at 24, and 48 hours post-infection (hpi) as stated. For drug treatment assays, infections were performed with co-treatment with camostat mesylate (Abcam), bafilomycin A1 (MedChemExpress) or E64d (MedChemExpress) and the culture supernatants were collected at 48 hpi for determination of viral copy number. Infection of human iPSC-CMs at a MOI of 0.1 was performed for determination of viral replication kinetics. The culture supernatants were collected at 24, 48, and 72 hpi for viral replication kinetics.

Viral titration by plaque assay
The viral culture supernatants were collected at designated time points, they were stored in -80ºC until viral titration was performed. The levels of infectious viral particles in our experiments were determined by plaque assay. Briefly, a confluent 12-well tissue culture plates of Vero E6/TMPRSS2 cells were prepared one day before the virus titration assay. Cells were washed with PBS once and incubated in 1ml PBS. 10-fold serial dilution of samples were performed, and the diluted samples were inoculated on the prepared Vero E6/TMPRSS2 cells with PBS, which was followed by 1 hour incubation at 37ºC in 5% CO_2. After the incubation, the supernatant was removed and was replenished with 2ml DMEM medium with 5% FBS and 1% agarose. The plates were then allowed for a further 48-72 hours incubation at 37ºC. After 48-72 hours, 10% formalin was added to the plates for an overnight fixation. The gels and formalin were discarded and 1% crystal violet in 20% ethanol was applied to the plates. The plaques were visualized after washing with tap water and the numbers of plaque were recorded.

Determination of viral copy number
Viral RNA was extracted using the QIAamp RNA Viral kit (Qiagen) according to the manufacturer's instructions. Extracted RNA was reverse transcribed to cDNA using PrimeScript RT Reagent Kit with gDNA Eraser (Takara) according to manufacturer's instruction. RT–qPCR was performed using TB Green Premix Ex Taq (Takara). The PCR program consisted of a pre-incubation for 10 min at 95 °C, followed by 40 cycles of denaturation for 10 sec at 95 °C, annealing for 10 sec at 60 °C and extension for 10 sec at 72 °C. For quantitation, 10-fold serial dilutions of standard plasmid equivalent to 10^{11}–10^{4} copies per reaction were prepared to generate the calibration curve. Real-time qPCR experiments were performed using LightCycler® 384 system (Roche, USA).

Immunofluorescence staining
After the infection or mock treatment, hiPSC-CMs were fixed with 4% paraformaldehyde in PBS for 30 minutes at room temperature. Permeabilization was performed by treating with 0.25% Triton X-100 in PBS for 15 minutes at room temperature. This was followed by a blocking step with 5% bovine serum albumin in PBS for 1 hour at room temperature. The samples were then incubated overnight at 4ºC with the primary antibody in the blocking medium. In the next day, incubation with secondary antibodies was performed in the dark at room temperature for 1 hour after series of washing steps. The samples were mounted with ibidi mounting medium (ibidi USA Inc., Fitchburg, WI). Fluorescence images were taken using a Leica THUNDER Imager microscope. A detailed list of antibodies for immunofluorescence staining is shown in additional file 3, Table S2.

Image analysis
Fluorescence images were processed with Fiji[4] and Leica LAS X software. Counting of cells positive for NP positive cells and cleaved caspase 3 and nuclei phenotypes were performed with CellProfiler 4.2.1[5]. Nuclei were counterstained with DAPI. Cells with nuclei within close proximity of each other were defined as multi-nucleated. At least 4,000 cells were counted for each treatment. Mitochondrial fragmentation was scored by a technician blinded to the identities of images. At least 200 cells were scored for each treatment. Scoring standards are shown in Figure S5.

Measurements of mitochondrial redox activity
Resazurin-based PrestoBlue assay (Thermo Fisher Scientific) was used for measuring mitochondrial redox activity of CMs according to the manufacturer's instructions. Ten-fold diluted Presto blue reagent was add to CMs for 60 mins at 37ºC. Relative fluorescence intensity with excitation and emission wavelengths of 540 nm and 590 nm, respectively was measured using FLUOstar OPTIMA plate-reader (BMG LABTECH, Ortenberg, Germany).

Hamster study
Eight to ten weeks old male golden Syrian hamsters were obtained from the laboratory animal services centre, Chinese University of Hong Kong via the Centre for Comparative Medicine Research at the University of Hong Kong (HKU). Hamsters were housed in ventilated isolator cages (IsoCage N, Tecniplast) at a temperature of 21°C, the humidity of 70%, and 12:12 dark/light cycles, with access to food and water ad libitum. All experiments followed the approved operating procedures of the Biosafety Level-3 animal facility at the LKS Faculty of Medicine, HKU. The animal study was approved by the Committee on the Use of Live Animals in Teaching and Research of the HKU.

Hamsters were anaesthetised with ketamine (150 mg/kg) and xylazine (10 mg/mg) via intraperitoneal injection and then intranasally challenged with 10^4 PFU (50 µl) of viruses or with PBS mock control. All hamsters were euthanized at 2 and 7 dpi by intraperitoneal injection of pentobarbital at 200 mg/kg.

Tissue sections were fixed in 4% paraformaldehyde and then processed for paraffin or OCT compound embedding. 5 µm paraffin sections were stained with haematoxylin and eosin for histopathological examination. Immunohistology on paraffin sections was performed using mouse anti-coronavirus nucleocapsid (NP) antibody. Immunofluorescence on 5 µm cryosections was performed using anti-NP and anti-MLC2V antibodies. A detailed list of antibodies for immunofluorescence staining is shown in additional file 3, Table S2. Hamster heart tissues were homogenized in Trizol reagent using plastic pestles. Total RNA from the lyzed samples were then isolated and purified using ReliaPrep™ RNA Miniprep Systems (Promega) following manufacturer's instructions. Total RNA integrity was checked using a 2100 Bioanalyzer (Agilent Technologies). Messenger RNA was purified from total RNA using poly-T oligo-attached magnetic beads.

RNA extraction from infected hiPSC-CM
Total RNA of the infected hiPSC-CM samples were extracted using Trizol reagent (ThermoFisher Scientific, Waltham, MA), the extraction procedures were performed according to the manufacturer's instructions. cDNA was synthesized from the total RNA with PrimeScript RT Master Mix (Takara Bio Inc.).

Bioinformatics analysis of hiPSC-CM RNA-seq Data

Preparation of RNA sample library and RNA-seq were performed by Novogene. Quantified libraries were pooled and sequenced on Illumina platform. The raw read data produced by RNA-seq experiments were first assessed by FastQC for quality control. The clean reads after adapter trimming by Trimmomatic tool were be mapped to the hg19 human reference genome using TopHat2. The read counts at gene level against GENCODE annotation were extracted using the HTSeq tool from bam. After data normalization, differentially expressed genes (DEGs) relative to mock infection control were identified and visualized by DESeq2/R package. DEGs were defined as fold changes at least 2 relative to mock, with an adjusted p-value<0.05 with FDR correction.

Functional analysis of the DEGs was conducted using web-based software DAVID (https://david.ncifcrf.gov/) to identify significantly enriched Gene ontology (GO) biological processes and KEGG pathways.

Ingenuity Pathway Analysis (IPA)

A table containing ENSEMBL gene IDs, corresponding log2 fold changes, and adjusted p-values of the differentially expressed genes was used as input for the IPA software (Qiagen). The source of the organism was filtered by Homo Sapiens to generate human molecular interaction pathway. IPA core analysis was performed with default parameters, by using tools including upstream regulators and regulator effect networks. For upstream regulators, the p-value of overlap is calculated using Fisher's Exact Test, and significance is generally attributed to p-values < 0.01. Z-score is >0 if the pathway or upstream regulator is activated, while z-score is <0 when they are inhibited. To focus on how the variants affect genes important for cardiac function which were downregulated, we focus on downregulated genes for this analysis. The unique upstream regulators in each dataset are identified by using the compare function and comparison analysis in IPA. Networks were generated based on unique upstream regulators in Omicron BA.2 dataset with the following parameters: active interaction sources including text mining, databases, experiments, and co-expression; an interaction score >0.4 (medium confidence).

Western blotting

Denatured protein lysate was loaded on 10% SDS-polyacrylamide gels for size separation. It was then electrophoretically transferred onto PVDF membranes (Pall Corporation) and blocked with 5% skimmed milk for 1 hour. Overnight incubation of primary antibodies was carried out at 4°C. The membranes were washed with PBS-T the next day and were incubated with horseradish peroxidase-conjugated goat anti rabbit antibody (Cell Signaling Technology, Inc., Danvers, MA) for 1 hour. After multiple PBS-T washing steps, the membrane was then developed with SignalFire Elite ECL Reagent (Cell Signaling Technology, Inc., Danvers, MA) according to the manufacturer's instruction. A detailed list of antibodies used in western blotting is shown in additional file 3, Table S2.

Statistics

GraphPad PRISM 8 (GraphPad Software, San Diego CA, USA) was used for statistical analysis. All *in vitro* data sets were analysed with student's T-test, one-way ANOVA or two-way ANOVA followed by Tukey's multiple comparisons test, presented as mean±SEM (standard error of the mean). The number of biological replicates are indicated in the figure legend. Clinical data were analysed using the Mann Witney test, presented as median and interquartile range. Differences with p-value less than 0.05 were considered statistically

significant.

Supplementary Reference:

1. Kwok M, Lee C, Li HS, Deng R, Tsoi C, Ding Q, Tsang SY, Leung KT, Yan BP, Poon EN: **Remdesivir induces persistent mitochondrial and structural damage in human induced pluripotent stem cell-derived cardiomyocytes**. *Cardiovasc Res* 2022, **118**(12):2652-2664.
2. Tsoi C, Deng R, Kwok M, Yan B, Lee C, Li HS, Ma CHY, Luo R, Leung KT, Chan GC *et al*: **Temporal Control of the WNT Signaling Pathway During Cardiac Differentiation Impacts Upon the Maturation State of Human Pluripotent Stem Cell Derived Cardiomyocytes**. *Front Mol Biosci* 2022, **9**:714008.
3. Waas M, Weerasekera R, Kropp EM, Romero-Tejeda M, Poon EN, Boheler KR, Burridge PW, Gundry RL: **Are These Cardiomyocytes? Protocol Development Reveals Impact of Sample Preparation on the Accuracy of Identifying Cardiomyocytes by Flow Cytometry**. *Stem cell reports* 2019, **12**(2):395-410.
4. Schindelin J, Arganda-Carreras I, Frise E, Kaynig V, Longair M, Pietzsch T, Preibisch S, Rueden C, Saalfeld S, Schmid B *et al*: **Fiji: an open-source platform for biological-image analysis**. *Nature methods* 2012, **9**(7):676-682.
5. Stirling DR, Carpenter AE, Cimini BA: **CellProfiler Analyst 3.0: Accessible data exploration and machine learning for image analysis**. *Bioinformatics* 2021.
