# Supplementary material for: SARS-CoV-2 variants divergently infect and damage cardiomyocytes in vitro and in vivo
Source: Cell Biosci. 2024 Aug 2;14:101. doi: 10.1186/s13578-024-01280-y (PMC11297708; doi:10.1186/s13578-024-01280-y)
Supplement: Supplementary file 2 — Additional file 2: Table S1. Primers used for RT-qPCR. [file 13578_2024_1280_MOESM2_ESM.pdf]

Table S1. Primers used for RT-qPCR

Target gene	Forward (5' to 3')	Reverse (5' to 3')
**Viral genes**		
SARS-CoV-2 RdRp	CGCATACAGTCTTRCAGGCT	GTGTGATGTTGAWATGACATGGTC
**Golden Hamster genes**		
B2m	AAATCGAGCTGCTGAAGAAT	GGGCTCCTTCAGAGTTATGT
Myl2	TTAGTCCACATCATCACCCA	CCTGTTTATTTGCGCATGAC
Myh6	ATCTGCCTAGCTCCCTATCT	CATCTTGCCGCTCTTAAACT
Cox6a2	CCCAGAGTTCATCCCTTACC	GATTGACGTGAGGATTGTGG
Sdha	TGCACACTTTGTATGGAAGG	CCAAGGCAAAATACTCCACA
Atp2a2	AGTTGTAAGCAGCCAGATTG	GGCCTACGTTGTAACCATTT
Kcnj2	GCAATGTTCAGTTCGTCAAC	GAGCTATCAGCCAAAACACA
Scn5a	AGTCACCAACCATGAGAGAA	AAAGGTGAAAATGCTCCCTC
**Human genes**		
B2M	CCACTGAAAAAGATGAGTATGCCT	CCAATCCAAATGCGGCATCTTCA
TNNI3	CCTCAAGCAGGTGAAGAAGG	CAGTAGGCAGGAAGGCTCAG
MYL2	ACATCATCACCCACGGAGAAGAGA	ATTGGAACATGGCCTCTGGATGGA
MYH7	GAGGACAAGGTCAACACCCT	CGCACCTTCTTCTCTTGCTC
mt-ATP6	CAACAACCGACTAATCACCA	GGGTGGTTGGTGTAAATGAG
SDHA	GTTCGACCGGGGAATGGTC	CCGCACCTTGTAGTCTTCCC
SCN5A	AGAGGAGTCTCGCCACAAGT	GACCACCAACTTCACTCCCT
KCNJ2	CTTGGGAATTCTGGTTTGCT	TGACTCAGCTGACATCCACAC
CACNA1C	TTCGTCATCGTCACCTTTCAG	TGTACTGGTGCTGGTTCTTG
ATP2A2	CAATGGCGCTCTCTGTTCTA	ATCCTCAGCAAGGACTGGTT
TMPRSS2	CTGCGTGGAAAAACCTCTTA	CTGGTTTCACTAGGTCGTTG
