# Supplementary material for: SARS-CoV-2 variants divergently infect and damage cardiomyocytes in vitro and in vivo
Source: Cell Biosci. 2024 Aug 2;14:101. doi: 10.1186/s13578-024-01280-y (PMC11297708; doi:10.1186/s13578-024-01280-y)
Supplement: Supplementary file 3 — Additional file 3: Table S2. Antibodies used for immunostaining and western blotting. [file 13578_2024_1280_MOESM3_ESM.pdf]

Table S2. Antibodies used for Immunostaining and Western blotting

Antibody	Supplier	Catalog number	Dilution
SARS-CoV/SARS-CoV-2 Nucleocapsid	Sino-Biological	40143-MM05	1:200 (IHC)<br>1:50 (IF)<br>1:500 (ICC)
MLC2V	ProteinTech	10906-1-AP	1:100
TOM20	abcam	ab186735	1:200
Donkey anti-Mouse IgG(H+L), Alexa Fluor 555	Invitrogen	A31570	1:1000
Donkey anti-Rabbit IgG(H+L), Alexa Fluor 647	Invitrogen	A31573	1:1000
TMPRSS2	Sino-Biological	204314-T08	1:1000
Cleaved Caspase 3	Cell Signaling	9664s	1:400
GAPDH	Cell Signaling	2118S	1:1000
Goat anti-Rabbit, HRP	Cell Signaling	7074	1:1000
