# Supplementary material for: SARS-CoV-2 variants divergently infect and damage cardiomyocytes in vitro and in vivo
Source: Cell Biosci. 2024 Aug 2;14:101. doi: 10.1186/s13578-024-01280-y (PMC11297708; doi:10.1186/s13578-024-01280-y)
Supplement: Supplementary file 4 — Additional file 4: Fig. S1. Delta, Omicron BA.1, and BA.2 differentially infect hiPSC-CMs. Human iPSC-CMs were exposed to Delta, Omicron BA.1, or BA.2, followed by immunostaining for NP. (A) Percentage of NP+ hiPSC-CMs infected at an MOI of 1 and harvested at 48 hpi, n = 4. (B) Percentage of NP+ hiPSC-CMs infected at an MOI of 0.1 and harvested at 24 or 48 hpi, n = 3. (C) Fluorescence images of the infected hiPSC-CMs, NP in Red, DAPI in blue. Data are presented as mean ± SEM, n refer to the number of biological replicates. Scale bar = 500 μm. [file 13578_2024_1280_MOESM4_ESM.pdf]

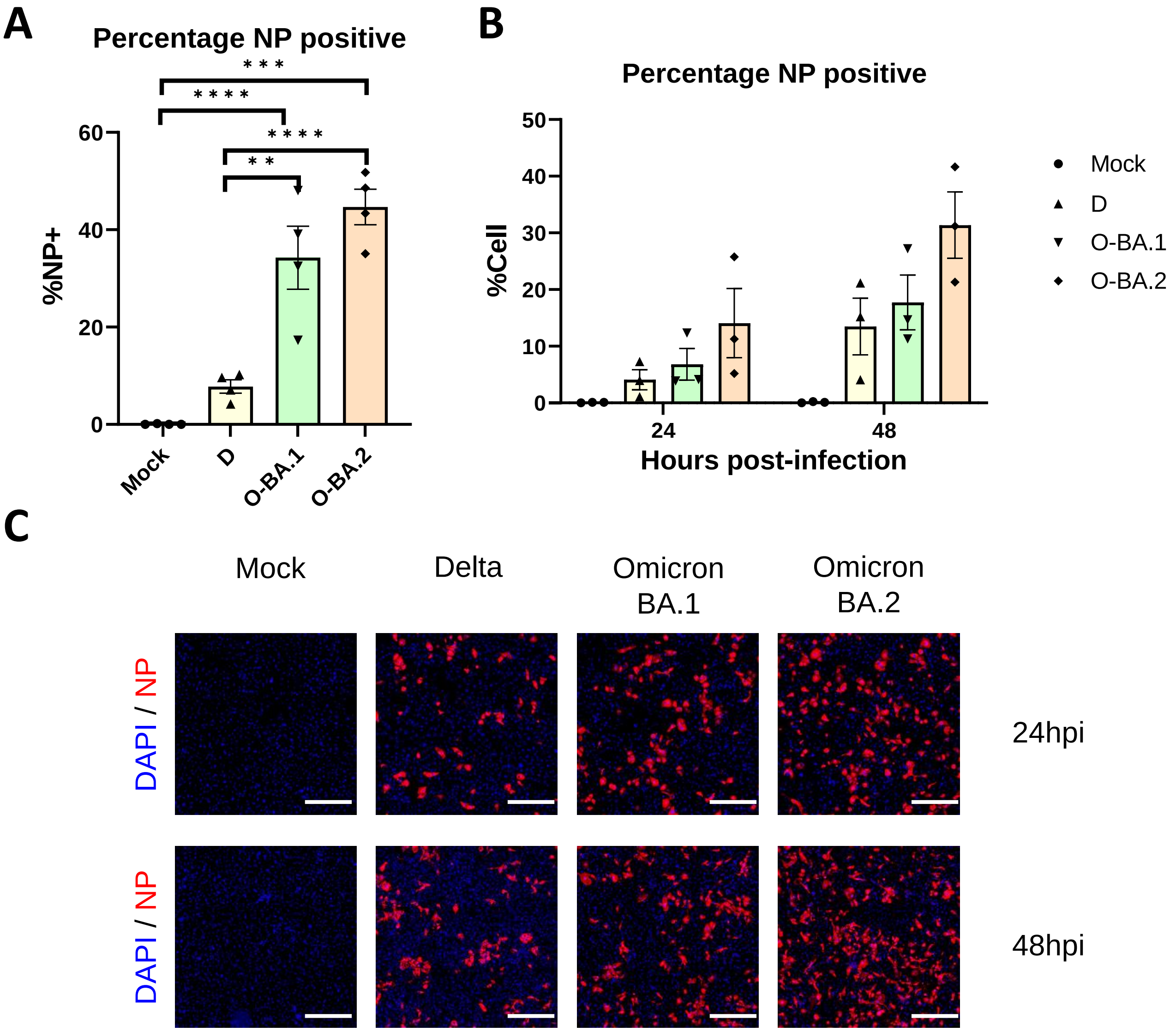

Fig.S1 Delta, Omicron BA.1, and BA.2 differentially infect hiPSC-CMs

Human iPSC-CMs were exposed to Delta, Omicron BA.1, BA.2. and were immunostained for NP. (**A**) Graph shows %NP^+ hiPSC-CMs at 48hpi with MOI of 1 n=4. (**B**) Graph shows %NP^+ hiPSC-CMs at 24 and 48hpi with MOI of 0.1 n=3. (**C**) Fluorescence images of infected hiPSC-CMs, NP in Red, DAPI in blue. Data are presented as mean±SEM, and n refer to biological replicates. Scale bar = 500µm.
