# Supplementary figures and images for: SARS-CoV-2 variants divergently infect and damage cardiomyocytes in vitro and in vivo
Source: Cell Biosci. 2024 Aug 2;14:101. doi: 10.1186/s13578-024-01280-y (PMC11297708; doi:10.1186/s13578-024-01280-y)

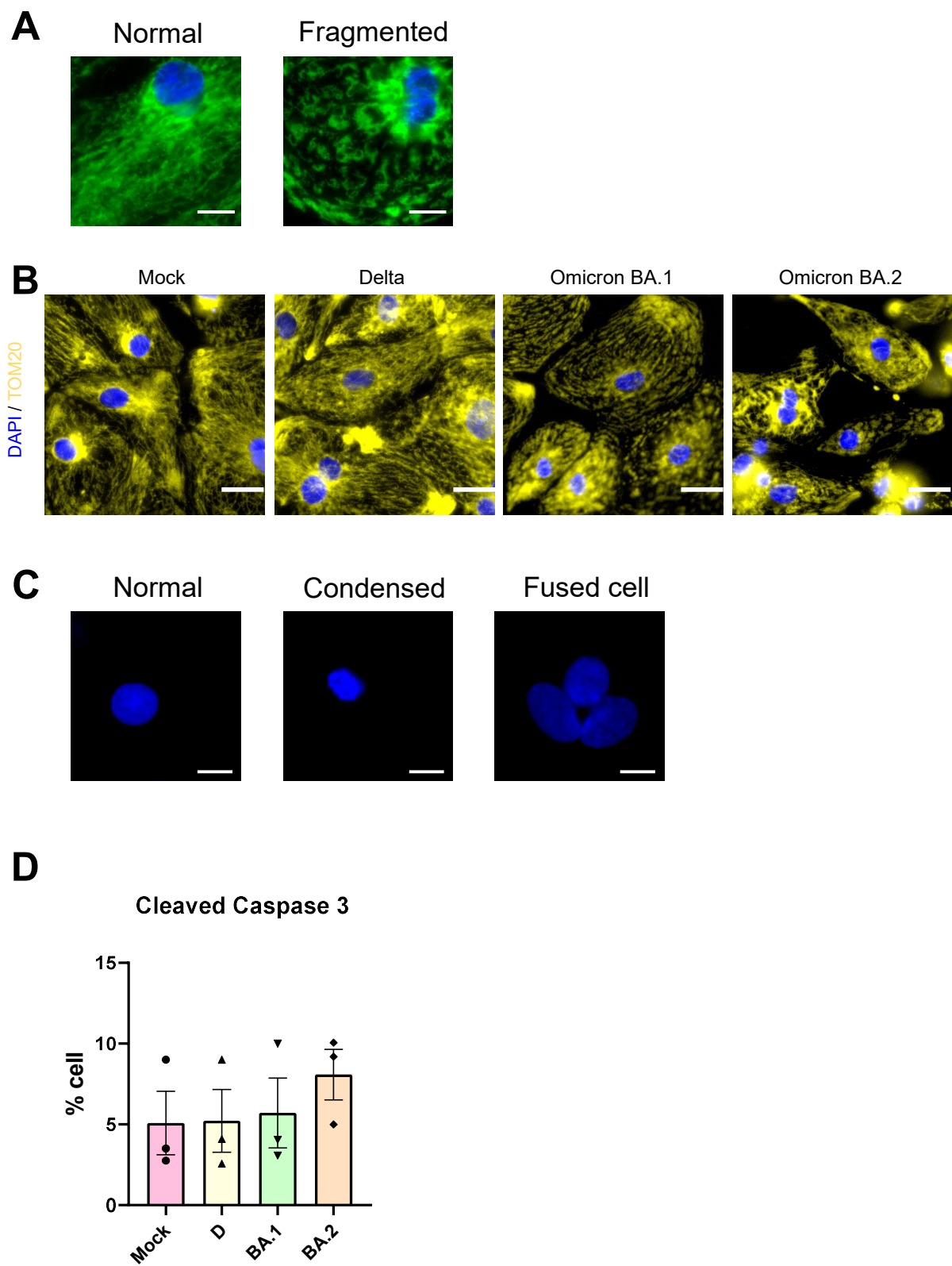

Fig.S2

Supplement: Supplementary file 5 — Additional file 5: Fig. S2. Omicron BA2 induces more severe damage in hiPSC-CMs. Human iPSC-CMs were infected with Delta (D), Omicron BA.1 (O-BA.1) or BA.2 (O-BA.2) for 48 h at an MOI of 1. (A) Representative images for scoring of mitochondrial fragmentation. Elongated, continuous mitochondria were scored as ‘Normal’, while punctate, discontinuous mitochondria are considered ‘Fragmented’. (B) Representative images of mock and infected hiPSC-CMs showing TOM20 immunostaining in yellow, DAPI nuclear staining in blue. (C) Representative images for scoring of nuclear condensation. Condensed nuclei are small, brightly stained, with irregular shapes. Multi-nucleated cells were defined as cells which contained more than one nucleus in close proximity of each other. (D) The percentage of hiPSC-CMs with cleaved caspase 3 staining was measured, n = 3. Scale bar A and B = 10 μm, C = 25 μm. [file 13578_2024_1280_MOESM5_ESM.pdf]

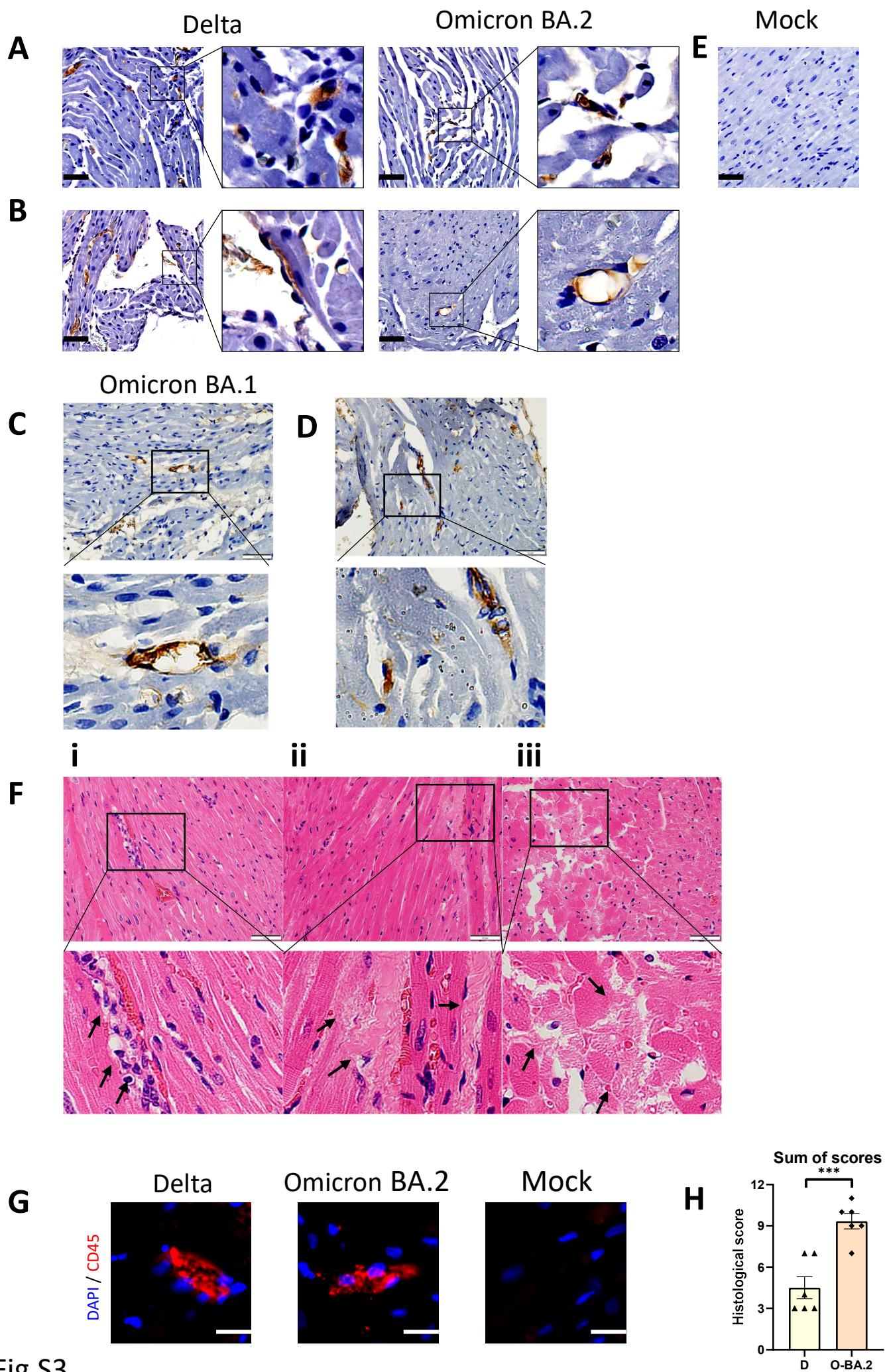

Fig.S3

Supplement: Supplementary file 6 — Additional file 6: Fig. S3. Infection of SARS-CoV-2 Delta and Omicron variants (BA.1 and BA.2 variants) in hamster heart. Groups of hamsters were inoculated intranasally with 104 PFU SARS-CoV-2 Delta, Omicron BA.1, BA.2 virus, or mock control. The hamsters were sacrificed at 2 dpi. Immunohistochemistry staining of SARS-CoV-2 nucleoprotein (NP) on heart sections were performed; positive cells were stained in brown color. Representative images of NP staining (A, C) Interstitial cells, (B, D) endothelial cells and (E) Mock, scale bar = 50 µm. (F) Representative images of H&E stained sections of Omicron BA.1 infected heart showing mild histopathological changes including perivascular immune cell accumulation (i, arrows), degenerated cardiomyocytes (ii, arrows) and a few foci of cardiomyocytes necrotic changes (iii, arrows). (G) Cryosections of the heart were stained with antibodies against CD45 in red, and DAPI nuclear staining in blue, showing sign of interstitial immune cell infiltration, scale bar = 25 µm. (H) Sum of histological scores of heart sections of all four pathological features, with maximal possible score of 12. Data are presented as mean ± SEM. n = 6 biological replicates for A, B, E, G and H; n = 3 for C, D, F. Statistical significance was calculated using Student’s t-test **p < 0.01, ***p < 0.001. [file 13578_2024_1280_MOESM6_ESM.pdf]

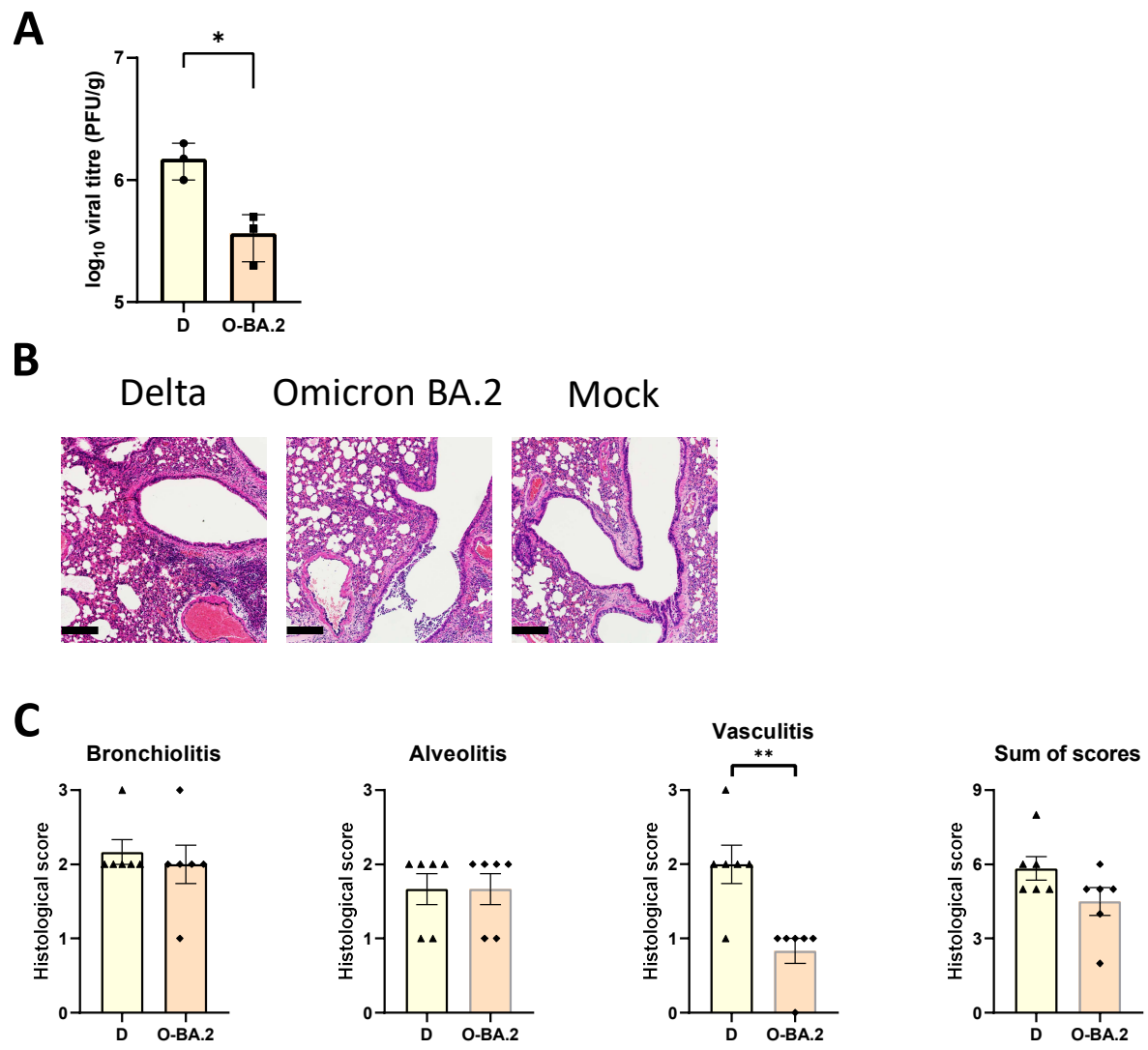

Fig.S4

Supplement: Supplementary file 7 — Additional file 7: Fig. S4. Infection of SARS-CoV-2 Delta and Omicron BA.2 variants in hamster lung. Groups of hamsters were inoculated intranasally with 104 PFU SARS-CoV-2 Delta, Omicron BA.2 virus, or mock control. The hamsters were sacrificed at 2 dpi; lung was fixed in formalin, processed to paraffin sections. (A) The viral titre in the lungs was determined using the plague assay. (n = 3) (B) H&E staining on hamster lung tissues were performed, representative images were shown. Mock control hamsters showed normal histological structures. Omicron BA.2 infected hamster lung showed bronchiolar epithelial cell damage with cell debris filled the lumen. Immune cell infiltration in the area surrounding the bronchiole and the vasculatures. Similar histological changes were also shown in Delta virus infected lung. Scale bar = 200 µm. (C) Histological scores of pathological features in the lungs, scaled 0–3, where 0 indicates the absence of pathological changes. Sum of histological scores of all three pathological features, with maximal possible score of 9. Data are presented as mean ± SEM, n = 6 biological replicates unless otherwise indicated. Statistical significance was calculated using Student’s t-test **p < 0.01, ***p < 0.001. [file 13578_2024_1280_MOESM7_ESM.pdf]

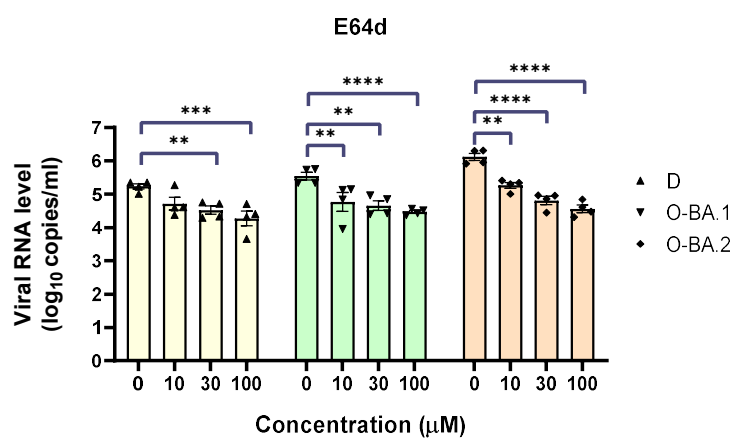

Fig.S5

Supplement: Supplementary file 9 — Additional file 9: Fig. S5. SARS-CoV-2 infection is inhibited by endocytosis inhibitor. Human iPSC-CMs were infected with Delta (D), Omicron BA.1 (O-BA.1) or BA.2 (O-BA.2) for 48 h at an MOI of 1, treated with E64d at the indicated concentrations. Viral RNA levels of SARS-CoV-2 variants were determined by qPCR analysis. Data are presented as mean ± SEM, n = 4 biological replicates. Significance is calculated in respect to the control group (0 µM); **p < 0.01, ***p < 0.001, ****p < 0.0001. [file 13578_2024_1280_MOESM9_ESM.pdf]
