# Supplementary material for: SARS-CoV-2 variants divergently infect and damage cardiomyocytes in vitro and in vivo
Source: Cell Biosci. 2024 Aug 2;14:101. doi: 10.1186/s13578-024-01280-y (PMC11297708; doi:10.1186/s13578-024-01280-y)
Supplement: Supplementary file 8 — Additional file 8: Table S3. A. Biological processes and pathways significantly enriched among DEGs commonly up- or down-regulated by all three variants in hiPSC-CMs. Table S3B. Biological processes and pathways significantly enriched among DEGs uniquely altered by Omicron BA.2. [file 13578_2024_1280_MOESM8_ESM.pdf]

Table S3A. Biological processes and pathways significantly enriched among DEGs commonly up- or down-regulated by all three variants in hiPSC-CMs

**Function categories**	**P value (log)**	**No. of DEGs**
Up-regulated DEGs		
hsa04010:MAPK signaling pathway	10.02	53
GO:0032922~circadian regulation of gene expression	7.90	20
GO:0006915~apoptotic process	6.32	67
GO:0006954~inflammatory response	5.47	49
hsa04668:TNF signaling pathway	4.25	21
hsa04064:NF-kappa B signaling pathway	4.20	20
hsa04115:p53 signaling pathway	4.03	16
GO:0034605~cellular response to heat	4.69	13
GO:0001525~angiogenesis	4.40	32
GO:0071456~cellular response to hypoxia	4.21	21
GO:0042981~regulation of apoptotic process	3.80	30
GO:0007249~I-kappaB kinase/NF-kappaB signaling	3.65	13
Down-regulated DEGs		
GO:0051301~cell division	12.04	42
GO:0007059~chromosome segregation	9.13	17
GO:0006260~DNA replication	7.01	18
GO:0006281~DNA repair	6.80	28
GO:0007052~mitotic spindle organization	6.18	12
hsa04110:Cell cycle	5.13	15
GO:0007049~cell cycle	4.69	27

Table S3B. Biological processes and pathways significantly enriched among DEGs uniquely altered by Omicron BA.2

**Function categories**	**P value (log)**	**No. of DEGs**
Up-regulated DEGs		
GO:0006357~regulation of transcription from RNA polymerase II promoter	10.56	121
GO:0006355~regulation of transcription, DNA-templated	9.06	78
hsa05168:Herpes simplex virus 1 infection	7.78	52
hsa05205:Proteoglycans in cancer	3.22	21
Down-regulated DEGs		
hsa01100:Metabolic pathways	20.20	176
GO:0032981~mitochondrial respiratory chain complex I assembly	9.11	19
GO:0006979~response to oxidative stress	7.82	24
hsa00190:Oxidative phosphorylation	7.27	27
hsa00020:Citrate cycle (TCA cycle)	2.83	8
