# Supplementary material for: SARS-CoV-2 variants divergently infect and damage cardiomyocytes in vitro and in vivo
Source: Cell Biosci. 2024 Aug 2;14:101. doi: 10.1186/s13578-024-01280-y (PMC11297708; doi:10.1186/s13578-024-01280-y)
Supplement: Supplementary file 10 — Additional file 10: Table S4. In vitro studies of SARS-CoV-2 using hPSC-CM models. [file 13578_2024_1280_MOESM10_ESM.pdf]

Table S4 *In vitro* studies of SARS-CoV-2 using hPSC-CM models

Author (Month Year)	Variant used	Title
Sharma, A. (Jul 2020)[1]	SARS-CoV-2/human/USA/WA1/2020	Human iPSC-Derived Cardiomyocytes Are Susceptible to SARS-CoV-2 Infection
Wong, C.K. (Sep 2020)[2]	Virus information not available	Human-Induced Pluripotent Stem Cell-Derived Cardiomyocytes Platform to Study SARS-CoV-2 Related Myocardial Injury
Bojkova, D. (Dec 2020)[3]	SARS-CoV-2/1/Human/2020/Frankfurt	SARS-CoV-2 infects and induces cytotoxic effects in human cardiomyocytes
	SARS-CoV-2/2/Human/2020/Frankfurt	
Li, Y. (Mar 2021)[4]	SARS-CoV-2/human/USA/WA1/2020	SARS-CoV-2 induces double-stranded RNA-mediated innate immune responses in respiratory epithelial-derived cells and cardiomyocytes
Marchiano, S. (Mar 2021)[5]	SARS-CoV-2/human/USA/WA1/2020	SARS-CoV-2 Infects Human Pluripotent Stem Cell-Derived Cardiomyocytes, Impairing Electrical and Mechanical Function
Bailey, A.L. (Apr 2021)[6]	SARS-CoV-2/human/USA/WA1/2020	SARS-CoV-2 Infects Human Engineered Heart Tissues and Models COVID-19 Myocarditis
Mills, R.J. (Apr 2021)[7]	hCoV-19/Australia/QLD02/2020	BET inhibition blocks inflammation-induced cardiac dysfunction and SARS-CoV-2 infection
Perez-Bermejo, J.A. (Apr 2021)[8]	SARS-CoV-2/human/USA/WA1/2020	SARS-CoV-2 infection of human iPSC–derived cardiac cells reflects cytopathic features in hearts of patients with COVID-19
Williams, T.L. (Jul 2021)[9]	SARS-CoV-2/human/Liverpool/REMRQ0001/2020	Human embryonic stem cell-derived cardiomyocyte platform screens inhibitors of SARS-CoV-2 infection
Yang, L. (Sep 2021)[10]	SARS-CoV-2/human/USA/WA1/2020	Cardiomyocytes recruit monocytes upon SARS-CoV-2 infection by secreting CCL2
Navaratnarajah, C.K. (Dec 2021)[11]	SARS-CoV-2/UW-001/Human/2020/Wisconsin	Highly Efficient SARS-CoV-2 Infection of Human Cardiomyocytes: Spike

		Protein-Mediated Cell Fusion and Its Inhibition
Salerno, J.A. (Dec 2021)[12]	SARS-CoV-2/human/BRA/RJ01/2020	Inhibition of SARS-CoV-2 infection in human iPSC-derived cardiomyocytes by targeting the Sigma-1 receptor disrupts cytoarchitecture and beating
Nchioua, R. (Dec 2022)[13]	BetaCoV/Netherlands/01/NL/2020	Strong attenuation of SARS-CoV-2 Omicron BA.1 and increased replication of the BA.5 subvariant in human cardiomyocytes
	hCoV-19/Netherlands/NH-EMC-1720/2021	
	hCoV-19/USA/CO-CDPHE-2102544747/2021	
	B.1.617.2 (Delta)	
	B.1.1.529, BA.5 (Omicron BA.5)	

Reference:

1. Sharma A, Garcia G, Jr., Wang Y, Plummer JT, Morizono K, Arumugaswami V, Svendsen CN: **Human iPSC-Derived Cardiomyocytes Are Susceptible to SARS-CoV-2 Infection**. *Cell Rep Med* 2020, **1**(4):100052.
2. Wong CK, Luk HK, Lai WH, Lau YM, Zhang RR, Wong AC, Lo GC, Chan KH, Hung IF, Tse HF *et al*: **Human-Induced Pluripotent Stem Cell-Derived Cardiomyocytes Platform to Study SARS-CoV-2 Related Myocardial Injury**. *Circulation journal : official journal of the Japanese Circulation Society* 2020, **84**(11):2027-2031.
3. Bojkova D, Wagner JUG, Shumliakivska M, Aslan GS, Saleem U, Hansen A, Luxan G, Gunther S, Pham MD, Krishnan J *et al*: **SARS-CoV-2 infects and induces cytotoxic effects in human cardiomyocytes**. *Cardiovasc Res* 2020, **116**(14):2207-2215.
4. Li Y, Renner DM, Comar CE, Whelan JN, Reyes HM, Cardenas-Diaz FL, Truitt R, Tan LH, Dong B, Alysandratos KD *et al*: **SARS-CoV-2 induces double-stranded RNA-mediated innate immune responses in respiratory epithelial-derived cells and cardiomyocytes**. *Proc Natl Acad Sci U S A* 2021, **118**(16).
5. Marchiano S, Hsiang TY, Khanna A, Higashi T, Whitmore LS, Bargehr J, Davaapil H, Chang J, Smith E, Ong LP *et al*: **SARS-CoV-2 Infects Human Pluripotent Stem Cell-Derived Cardiomyocytes, Impairing Electrical and Mechanical Function**. *Stem cell reports* 2021, **16**(3):478-492.
6. Bailey AL, Dmytrenko O, Greenberg L, Bredemeyer AL, Ma P, Liu J, Penna V, Winkler ES, Sviben S, Brooks E *et al*: **SARS-CoV-2 Infects Human Engineered Heart Tissues and Models COVID-19 Myocarditis**. *JACC Basic Transl Sci* 2021, **6**(4):331-345.
7. Mills RJ, Humphrey SJ, Fortuna PRJ, Lor M, Foster SR, Quaife-Ryan GA, Johnston RL, Dumenil T, Bishop C, Rudraraju R *et al*: **BET inhibition blocks inflammation-induced cardiac dysfunction and SARS-CoV-2 infection**. *Cell* 2021, **184**(8):2167-2182 e2122.
8. Perez-Bermejo JA, Kang S, Rockwood SJ, Simoneau CR, Joy DA, Silva AC, Ramadoss GN, Flanigan WR, Fozouni P, Li H *et al*: **SARS-CoV-2 infection of human iPSC-derived cardiac cells reflects cytopathic features in hearts of patients with COVID-19**. *Sci Transl Med* 2021, **13**(590).
9. Williams TL, Colzani MT, Macrae RGC, Robinson EL, Bloor S, Greenwood EJD, Zhan JR,

Strachan G, Kuc RE, Nyimanu D *et al*: **Human embryonic stem cell-derived cardiomyocyte platform screens inhibitors of SARS-CoV-2 infection**. *Commun Biol* 2021, **4**(1):926.

10. Yang L, Nilsson-Payant BE, Han Y, Jaffre F, Zhu J, Wang P, Zhang T, Redmond D, Houghton S, Moller R *et al*: **Cardiomyocytes recruit monocytes upon SARS-CoV-2 infection by secreting CCL2**. *Stem cell reports* 2021, **16**(9):2274-2288.
11. Navaratnarajah CK, Pease DR, Halfmann PJ, Taye B, Barkhymer A, Howell KG, Charlesworth JE, Christensen TA, Kawaoka Y, Cattaneo R *et al*: **Highly Efficient SARS-CoV-2 Infection of Human Cardiomyocytes: Spike Protein-Mediated Cell Fusion and Its Inhibition**. *J Virol* 2021, **95**(24):e0136821.
12. Salerno JA, Torquato T, Temerozo JR, Goto-Silva L, Karmirian K, Mendes MA, Sacramento CQ, Fintelman-Rodrigues N, Souza LRQ, Ornelas IM *et al*: **Inhibition of SARS-CoV-2 infection in human iPSC-derived cardiomyocytes by targeting the Sigma-1 receptor disrupts cytoarchitecture and beating**. *PeerJ* 2021, **9**:e12595.
13. Nchioua R, Diofano F, Noettger S, von Maltitz P, Stenger S, Zech F, Munch J, Sparrer KMJ, Just S, Kirchhoff F: **Strong attenuation of SARS-CoV-2 Omicron BA.1 and increased replication of the BA.5 subvariant in human cardiomyocytes**. *Signal Transduct Target Ther* 2022, **7**(1):395.
